# (±)-Rutacycoumarins A and B: two pairs of unprecedented coumarin enantiomers from the aerial part of *Ruta graveolens* L. and chemically synthesized

**DOI:** 10.1080/13880209.2025.2600291

**Published:** 2025-12-13

**Authors:** Xu Feng, Jing Fang, Yanyang Liu, Chengyu Ge, Xiaolin Liao, Tao Jiang, Xiongjun Hou, Hao Huang, Shao Liu, Aimin Wang, Yueping Jiang

**Affiliations:** ^a^ Department of Pharmacy, Xiangya Hospital, Central South University, Changsha, China; ^b^ National Clinical Research Center for Geriatric Disorders, Xiangya Hospital, Central South University, Changsha, China; ^c^ Department of Clinical Pharmacy, Hunan University of Medicine General Hospital, Huaihua, China; ^d^ Department of Emergency, Hunan University of Medicine General Hospital, Huaihua, China; ^e^ Department of Pharmacy, Jiangxi Provincial People’s Hospital, the First Affiliated Hospital of Nanchang Medical College, Nanchang, China; ^f^ Jiangxi Province Key Laboratory of Pharmacology of Traditional Chinese Medicine, School of Pharmacy, Gannan Medical University, Ganzhou, China; ^g^ State Key Laboratory Neurology and Oncology Drug Development, Nanjing, China; ^h^ Department of Emergency, Xiangya Hospital, Central South University, Changsha, China

**Keywords:** *Ruta graveolens* L., coumarin enantiomers, monoamine oxidase B inhibitory activity, antihepatitic activity

## Abstract

**Context:**

Benzofuran derivatives are important structural motifs found in natural products, often exhibiting significant biological activities. *Ruta graveolens* L. is a plant source known for containing diverse bioactive compounds.

**Objective:**

This study aimed to isolate and characterize compounds from the aerial parts of *R. graveolens*, confirm their structures through synthesis, develop a novel synthetic methodology, and evaluate their potential anti-inflammatory effects and monoamine oxidase inhibitory activity.

**Materials and methods:**

The structures of benzofuran enantiomers were elucidated using integrated NMR, HRMS, and ECD analyses. (±)-Rutacycoumarins A and B were synthesized *via* a novel direct C3 alkylation of coumarin bearing phenolic hydroxyl groups. Biological evaluation assessed the anti-inflammatory effects of (±)-Rutacycoumarins A and B in LPS-stimulated HepG2 cells by measuring liver biomarkers and pro-inflammatory cytokines, and their inhibitory activity against monoamine oxidase B (MAO-B).

**Results:**

Two novel Z/E pairs of benzofuran enantiomers, (±)-Rutacycoumarins A and B, featuring fused cyclopropane motifs, were isolated and structurally confirmed. Their synthesis employed a novel catalytic EDA complex (DIPEA/potassium ethyl xanthate donor, NHPI ester acceptor). (±)-Rutacycoumarins A and B reduced liver biomarkers and pro-inflammatory cytokines in LPS-treated HepG2 cells, and all four enantiomers inhibited MAO-B.

**Conclusions:**

This study isolated two novel benzofuran enantiomer pairs with fused cyclopropane motifs from *R. graveolens* Their structures were confirmed *via* a new catalytic EDA complex synthesis. Racemic mixtures reduced LPS-induced liver/cellular damage and cytokines in HepG2 cells, while all enantiomers inhibited MAO-B.

## GRAPHICAL ABSTRACT

**Figure:**
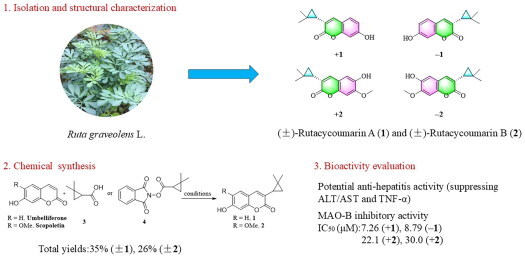

## Introduction

Natural products, particularly chemical constituents derived from traditional Chinese medicine (TCM), are a significant source for new drug discovery owing to their complex structural features and diverse pharmacological activities (Bai et al. 2024; Ma et al. 2024; Su et al. 2024; Tang et al. 2024; Zhang et al. 2024). *Ruta graveolens* L. (family Rutaceae), native to the Mediterranean region, is a medicinal plant cultivated worldwide, spanning Europe and numerous Asian countries (Pollio et al. 2008). Historically utilized in traditional Chinese medicine (e.g., *Compendium of Materia Medica*), *R. graveolens*, known as ‘stinky grass’, due to its pungent odor-has been employed for hemostasis, blood stasis resolution, and anti-inflammatory therapies (Ainiwaer et al., 2022). Modern phytochemical studies have identified diverse bioactive constituents, including alkaloids (Salib et al. 2014; Wang et al. 2024), coumarins (Raghav et al. 2007; Mancuso et al. 2015; Ainiwaer et al., 2022), and terpenoids (Szewczyk et al. 2022; Ainiwaer et al. 2023; Shahrajabian 2023), which underpin their antibacterial (Pavić et al. 2019; Liu et al. 2023; Makevych et al. 2023), anti-inflammatory (Raghav et al. 2006; Mokhtar et al. 2022), antioxidant (Ratheesh et al. 2009; Cefali et al. 2019; Ainiwaer et al. 2023), anticancer (Schelz et al. 2016), antiproliferative (Schelz et al. 2016), reproductive regulation, and antifungal (Peralta-Ruiz et al. 2020) properties. The biological activities of these phytoconstituents are strongly influenced by the type and quantity of secondary metabolites, which in turn are affected by environmental and genetic factors. Variations in soil composition, climate, and growth stage can lead to substantial differences in metabolite profiles, consequently altering the bioactivity of *R. graveolens* extracts (Özay 2025).

Neurodegenerative disorders, such as Parkinson’s disease (PD), represent a pervasive global health burden (Bloem et al. 2021; Zhu et al. 2023). Notably, monoamine oxidase B-induced oxidative stress, a key enzymatic driver of PD progression B (MAO-B)-induced oxidative stress have emerged as a critical therapeutic target. Natural coumarins (Stefanachi et al. 2018), known for their bioactive structural scaffolds, are emerging as promising candidates for neurodegenerative therapeutic research. Hepatic diseases continue to be one of the most significant risks to public health and remain a major health concern worldwide because of the multiple functions conducted/performed by the liver (Datta et al. 2023). It is a valuable strategy to explore traditional Chinese medicine and natural drugs for hepatoprotective agents (Lam et al. 2016; Datta et al. 2023). Our previous study revealed that graveoline, a phenylquinolin alkaloid in *R. graveolens*, is a hepatoprotective agent that attenuates liver injury by suppressing TNF-α/JAK1/STAT3 signaling while enhancing IL-4/IL-10 production, highlighting the potential of *R. graveolens* as a source for anti-hepatitic agents (He et al. 2024).

**Figure 1.**
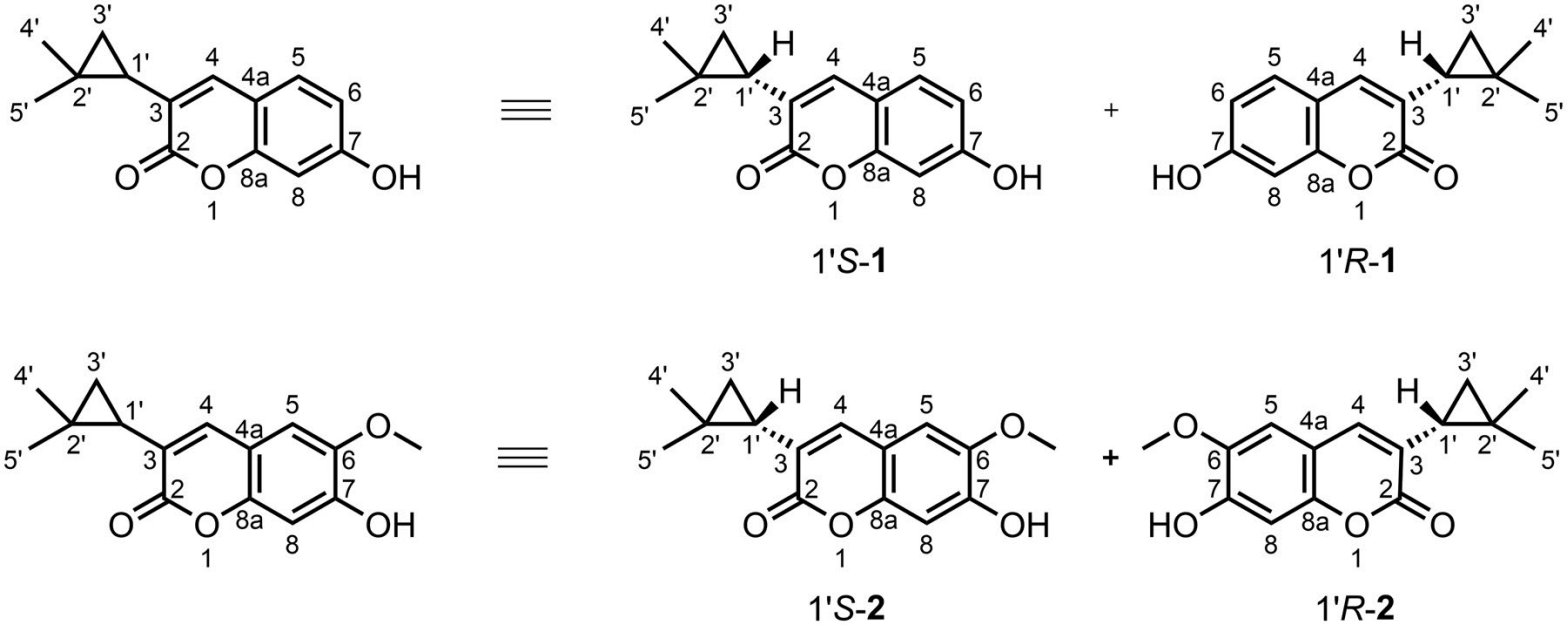
The structure of (±)-Rutacycoumarin A (**1**) and (±)-Rutacycoumarin B (**2**).

In this study, we meticulously detail the processes of isolation, structural identification, chemical synthesis, and exploration of the biological activities of four novel benzofuran compounds. These compounds, (±)-Rutacycoumarin A (**1**) and (±)-Rutacycoumarin B (**2**), were derived from *R. graveolens* (Figure 1. We then evaluated the anti-hepatitis activity of racemic mixtures (±)-**1** and (±)-**2**, demonstrating their protective effects against lipopolysaccharide (LPS)-induced liver injury through significant suppression of alanine transaminase (ALT), aspartate transaminase (AST), and pro-inflammatory cytokines. Notably, (±)-**1** and (±)-**2** also potently inhibited MAO-B, a key therapeutic target in Parkinson’s, highlighting their dual pharmacological potential.

## Material and methods

### General experimental procedures

Optical rotations were acquired on an AUTOPOL II automatic polarimeter with a micro polarizer of 10 mm path length (Rudolph research analytical, USA). UV spectra were recorded on a Cary 300 UV-Vis spectrophotometer (Agilent Technologies Co., Ltd. USA), while circular dichroism (CD) spectra were acquired on a JASCO J-815 spectrometer (JASCO Corporation, Japan). Infrared (IR) spectra were obtained using a Nicolet IS50 FT-IR spectrometer (Thermo Fisher Scientific Co. Ltd., USA). NMR spectra, including 1D and 2D experiments, were acquired in CD_3_OD on Bruker AVANCE III 400 MHz and AVANCE NEO 600 MHz spectrometers (Bruker, Ltd, Germany), corresponding to magnetic field strengths of 9.4 T and 14.1 T, respectively. Chemical shifts are reported relative to tetramethylsilane (TMS) as an internal standard. High-resolution ESI-MS (HR-ESI-MS) data were collected using an Agilent 1290 HPLC system coupled to an Agilent G6545B Q-TOF mass spectrometer (Agilent Technologies Co., Ltd. USA). Chromatographic separation was achieved on a C18 column (2.1 × 100 mm, 1.8 μm; Agilent Technologies Co., Ltd. USA) with gradient elution optimized for each analyte. For preparative separation, HPLC was performed using an Agilent system comprising a G1311A Quat Pump and a G1315B DAD detector, equipped with a YMC Pack ODS-A semi-preparative column (250 mm × 10 mm, 5 μm, YMC Co. Ltd, Japan). Chiral separations were carried out on another Agilent system fitted with a 1260 Quit Pump VL, a G7114A VWD detector, and a fraction collector, using a CHIRALPAK AD-H column (0.46 cm I.D. × 25 cm, Daicel chemical industries Co. Ltd, Japan). Column chromatography was carried out using D101 macroporous resin (0.3–1.25 mm, Qingdao Marine Chemical, China) or silica gel (200–300 mesh, Qingdao Marine Chemical, China). Flash chromatography was performed on Sephadex LH-20. Thin-layer chromatography (TLC) was conducted on pre-coated silica gel GF254 plates (Qingdao Marine Chemical, China), with spots visualized under UV light (254/301 nm) or by spraying with 10%H_2_SO_4_ in EtOH (1: 9, v/v) followed by heating. All solvents used were of HPLC grade (Tedia Company Inc. USA) and filtered through 0.45 μm or 0.22 μm membranes prior to use.

### Isolation and identification of natural compounds

#### Plant material

Dried aerial parts of *R. graveolens* were collected from Guangning County (23°67’N, 112°20’E; Zhaoqing City, Guangdong Province, China) for experimental analysis. This plant is not protected, and the local government permits harvesting. Botanical authentication was performed by Prof. Shao Liu of Xiangya Hospital, Central South University, with voucher specimens (ID 2,020,001) archived in the Medicinal Chemistry Laboratory at Xiangya Hospital’s Pharmacy Building for long-term preservation and future. The Medicinal Chemistry Laboratory at the Xiangya Hospital Pharmacy Building is located on 87 Xiangya Road, Changsha 410008, China.

#### Extraction and isolation of compounds

The aerial parts of dried *R. graveolens* (10 kg) were mechanically ground and subjected to dual-phase ultrasonic-assisted extraction (8 L of ultrapure water × 1 h per cycle at 25 °C) using a Branson 5800 system. The combined filtrates were vacuum concentrated (Rotavapor R-300, Büchi; 40 °C, 15 mbar) to yield an aqueous extract (1,823.7 g). Primary fractionation employed D101 macroporous resin with sequential elution: 5 L H_2_O(A), 4 L 50% EtOH (B), and 5 L 90% EtOH (C). The ethanol-rich fraction (C) was lyophilized to obtain a dark-brown crude extract. Following solvent removal, fraction C (14.3 g) was subjected to silica gel chromatography using a methanol (0% to 100%) gradient in dichloromethane. The resulting YXC-1 to YXC-11 subfractions were identified using TLC. YXC-3 (2.5 g) was further purified by Sephadex LH-20 column chromatography using a solvent system consisting of petroleum ether, dichloromethane, and methanol (5:5:1). The resulting fractions were identified by TLC and combined to yield 10 fractions (YXC-3-1 to YXC-3-10). YXC-3-7 (0.18 g) was purified using a thin-layer preparative plate with a solvent system of petroleum ether: ethyl acetate (4:1), resulting in three fractions (YXC-3-7-1–YXC-3-7-3). YXC-3-7-5 (∼35 mg) was further purified using semi-preparative HPLC (Ph column, ACN-H_2_O-0.1% FA, v/v, 38:62, 2.0 mL/min), yielding compound **1** (4.6 mg, *t*_R_ = 34.0 min). YXC-3-7-2 (∼20 mg) was further purified using semi-preparative HPLC (Ph column, ACN-H_2_O-0.1% FA, v/v, 38:62, 2.0 mL/min), yielding compound **2** (4.3 mg, *t*_R_ = 32.3 min). Subsequent separation of **1** by semipreparative HPLC using a normal phase chiral column (CHIRALPAK AD-H) and eluting with n-hexane/isopropyl alcohol (9:1, 0.7 mL/min) afforded compound (–)-**1** (2.0 mg, *t*_R_ = 10.721 min) and compound (+)-**1** (2.1 mg, *t*_R_ = 19.197 min), respectively. Similarly, separation of **2** by HPLC using a normal-phase chiral column (CHIRALPAK AD-H) and elution with n-hexane/isopropyl alcohol (9:1, 0.7 ml/min) afforded compounds (+)-**2** (1.6 mg, *t*_R_ = 12.385 min) and (–)-**2** (1.7 mg, *t*_R_ = 16.028 min), respectively.

(+)-**1**: white amorphous powder, [*α*]25 *D* + 103.8 (*ⅽ* 0.025, MeOH); UV (MeOH) λ_max_ (log *ε*) = 330 (1.81) nm; CD (MeOH) λ_max_ = 211 (mdeg −5.298), 229 (mdeg +2.178), 254 (mdeg +1.438), 32 (mdeg −2.251) nm; IR (KBr) ν_max_ 3415, 3285, 2953, 2925, 2853, 1700, 1654, 1598, 1260, 1191, 1120, 1033, 862 cm^−1^;^1^H NMR (CD_3_OD, 500 MHz) and ^1^³C NMR (CD_3_OD, 125 MHz) spectroscopic data, see Table 1, HR-ESIMS *m/z* 231.1016 [M + H]^+^ (calcd for C_14_H_15_O_3_, 231.1016).

**Table 1.** NMR Spectral data (δ) for compounds (±)-**1** (500 MHz, 125 MHz) and (±)-**2** (600 MHz, 150 MHz) in CD3OD (δ in ppm, *J* in hz).

position	1			2	
	δ_H_ (H, *J* in Hz)	δ_C_		δ_H_ (H, *J* in Hz)	δ_C_
1	/	/	1	/	/
2	/	165.3	2	/	165.6
3	/	124.9	3	/	125.3
4	7.48 s (1H)	140.6	4	7.50 s (1H)	140.7
4a	/	113.5	4a	/	113.0
5	7.40 d (1H,9.0)	130.0	5	7.08 s (1H)	109.5
6	6.76 d (1H,9.0)	114.4	6	/	147.0
7	/	162.0	7	/	151.8
8	6.70 s (1H)	103.0	8	6.76 s (1H)	103.6
8a	/	155.9	8a	/	150.0
1′	1.72 t (1H,7.5)	26.7	1′	1.73 (1H,6.6)	26.7
2′	/	20.4	2′	/	20.5
3′	0.80 (2H, overlap)	18.2	3′	0.83 (2H,overlap)	18.2
4′	0.86 s (3H)	19.7	4′	0.86 s (3H)	19.7
5′	1.25 s (3H)	27.1	5′	1.26 s (3H)	27.1
			7-OCH_3_	3.90 s	56.8

(–)-**1**: white amorphous powder, [*α*]25 D −90.0 (*ⅽ* 0.036, MeOH); The UV and IR spectra of (–)-**1** exhibited characteristic absorptions same as those of (+)-**1**. CD (MeOH) λ_max_ = 211 (mdeg +4.251), 228 (mdeg −2.538), 252 (mdeg −1.176), 328 (mdeg +1.985) nm; ^1^H-NMR (CD_3_OD, 500 MHz) and ^1^³C-NMR (CD_3_OD, 125 MHz) spectroscopic data, see Table 1, HR-ESIMS *m/z* 231.1016 [M + H]^+^ (calcd for C_14_H_15_O_3_, 231.1016).

(+)-**2**: faint yellow amorphous powder, [*α*]25 *D* + 60.5 (*ⅽ* 0.096, MeOH); UV (MeOH) λ_max_ (log *ε*) = 345 (1.78) nm; CD (MeOH) λ_max_ = 219 (mdeg −4.391), 244 (mdeg −0.812), 262 (mdeg +1.861), 339 (mdeg −1.190) nm; IR (KBr) ν_max_ 3350, 2922, 2851, 1709, 1582, 1507, 1464, 1379, 1274, 1230, 1149, 1124, 1036 cm^−1^; ^1^H-NMR (CD_3_OD, 600 MHz) and ^1^³C-NMR (CD_3_OD, 150 MHz) spectroscopic data, see Table 1, HR-ESIMS *m/z* 261.1125 [M + H]^+^ (calcd for C_15_H_17_O_4_, 261.1122).

(–)-**2**: faint yellow amorphous powder, [*α*]25 D −51.0 (*ⅽ* 0.094, MeOH); The UV and IR spectra of (–)-**2** exhibited characteristic absorptions same as those of (+)-**2**. CD (MeOH) λ_max_ = 218 (mdge +4.496), 244 (mdeg +0.875), 263 (mdeg −1.866), 338 (mdeg +1.206) nm;^1^H-NMR (CD_3_OD, 600 MHz) and ^1^³C-NMR (CD_3_OD, 150 MHz) spectroscopic data, see Table 1, HR-ESIMS *m/z* 261.1125 [M + H]^+^ (calcd for C_15_H_17_O_4_, 261.1122).

#### ECD calculation

We performed theoretical electronic circular dichroism (ECD) calculations using time-dependent density functional theory (TDDFT) at the mPW1PW91/6–311G(d) level, with the IEF-PCM solvation model applied for methanol (MeOH). Conformational sampling for each diastereomer was first carried out using the MMFF94 force field, followed by further exploration of low-energy conformers *via* Crest at the GFNFF level. The resulting conformers were then optimized at the GFN2-XTB level, and those within a 4 kcal/mol energy window were retained for subsequent ECD calculations. For each conformer, the ECD spectrum was computed considering 20 excited states. The final Boltzmann-averaged ECD spectrum was generated using SpecDis v1.71, based on the Gibbs free energies of the conformers and applying a Gaussian broadening with a sigma/gamma value of 0.35 eV.

### Chemical synthesis

#### Synthesis of 1,3-dioxoisoindolin-2-yl 2,2-dimethylcyclopropane-1-carboxylate (4)

The 2,2-dimethylcyclopropanecarboxylic acid (1 g, 8.76 mmol), N, N-hydroxyphthalimide (1.57 g, 9.63 mmol), 4-dimethylaminopyridine (DMAP, 107.0 mg, 0.88 mmol), DIC(, 1.7 g, 9.63 mmol), and dichloromethane (8.8 ml) were added to an oven-dried flask with a magnetic stirring bar. The reaction mixture was stirred at room temperature for 3 h until complete. The solution was concentrated under reduced pressure to remove dichloromethane, and the residue was purified by recrystallization (diethyl ether) to obtain **4**. Compound **4** was used as received without further purification.

#### Synthesis of Rutacycoumarin A (1)

To a 10 mL Schlenk flask equipped with a magnetic stirring bar were added umbelliferone (1.0 mmol, 160 mg), 4 (1.0 mmol, 260 mg), Na_2_S (13 mg, 0.1 mmol), potassium ethyl xanthate (16 mg, 0.1 mmol), and dimethyl sulfoxide (DMSO) (2.5 mL). The resulting mixture was charged with N_2_ and irradiated with 365 nm LEDs (36 W) for 48 h. The reaction mixture was extracted with dichloromethane. The organic layer was dried over Na_2_SO_4_, filtered, and concentrated under reduced pressure. The residue was purified by silica gel flash chromatography to yield the corresponding product Rutacycoumarin A (**1**) (81 mg, 35%).^1^H NMR (500 MHz, CD_3_OD) *δ*_H_ 7.48 (s, 1H), 7.40 (d, *J* = 8.5 Hz, 1H), 6.77 (dd, *J* = 8.5, 2.3 Hz, 1H), 6.71 (d, *J* = 2.1 Hz, 1H), 1.73 (t, *J* = 7.1 Hz, 1H), 1.27 (s, 3H), 0.87 (s, 3H), 0.81 (dt, *J* = 8.0, 5.1 Hz, 2H).^13^C NMR (100 MHz, CD_3_OD) (*δ*_C_ 165.3, 162.0, 155.8, 140.6, 129.9, 124.9, 114.3, 113.5, 103.0, 27.0, 26.6, 20.4, 19.7, 18.1). These data are consistent with the separated spectral data (Figures S14 and S15 in the supporting information).

#### Synthesis of Rutacycoumarin B (2)

To a 10 mL Schlenk flask equipped with a magnetic stirring bar were added scopoletin (0.1 mmol, 20 mg), 4 (0.1 mmol, 26 mg), Na_2_S (1.3 mg, 0.01 mmol), potassium ethyl xanthate (1.6 mg, 0.01 mmol), and DMSO (0.25 mL) were added to a 10 mL Schlenk flask equipped with a magnetic stirring bar. The resulting mixture was charged with N_2_ and irradiated with 365 nm LEDs (36 W) for 48 h. The reaction mixture was extracted with dichloromethane. The organic layer was dried over Na_2_SO_4_, filtered, and concentrated under a reduced pressure. The residue was purified using silica gel flash chromatography to obtain the corresponding product Rutacycoumarin B (**2**) (6.8 mg, 26%). ^1^H NMR (400 MHz, CD_3_OD) [*δ*_H_ 7.50 (1H,s), 7.08 (1H,s), 6.77 (1H,s), 3.91 (1H,s), 1.74 (1H, *J* = 7.6, 6.2, 1.0 Hz, ddd), 1.27 (3H,s), 0.87 (3H,s), 0.81 (2H, *J* = 8.1, 5.0 Hz, dt)]. ^13^C NMR (100 MHz, CD_3_OD) *δ*_C_ (165.6, 151.7, 149.9, 147.0, 140.7, 125.2, 112.9, 109.5, 103.6, 56.8, 27.0, 26.7, 20.4, 19.7, 18.2). These data are consistent with the separated spectral data (Figures S27 and S28 in the supporting information).

### Biological evaluation

#### Recombinant human MAO-B enzyme inhibition assay

Test compounds were prepared by 3-fold serial dilution across 10 concentrations, starting from 10 μM, with R(−)-deprenyl included as a positive control. All compounds were tested with technical replicates. Compounds (100× final concentration) in 384-well source plates were dispensed (200 nL per well) into assay plates. Min/Max control wells received 200 µL of 100% DMSO. All wells except the Min controls were supplemented with 10 μL of 10 nM MAO-B (Min controls received buffer instead). Plates were centrifuged at 1000 × g for 1 min and incubated at 25 °C for 15 min. Then, 10 μL of 0.5 μM substrate was added to each well. After further centrifugation, the plates were protected from light and incubated for 60 min. The reaction was terminated by adding 20 μL of stop solution, followed by centrifugation and a 20-min equilibration period. Fluorescence was measured using an EnVision microplate reader. The inhibition percentage was calculated as follows: Inhibition (%) = [signal (max)− signal (sample)]/[signal (max) − signal (min)] × 100. Dose–response curves were generated by plotting the logarithm of compound concentration (X-axis) against the percentage inhibition (Y-axis). Curve fitting was performed in GraphPad Prism 8 using the ‘log(inhibitor) vs. response -Variable slope’ model to determine the IC_50_ values for MAO-B inhibition.

#### Cell culture and reagents

HepG2 cells were acquired from the American Type Culture Collection (ATCC, Homo sapiens, CVCL_0027, No. SCSP-510) and cultured in Minimum Essential Medium (MEM) supplemented with 10% fetal bovine serum (FBS, Gibco, No. 10099141), 100 U/mL penicillin, and 100 μg/mL streptomycin. Cells were maintained at 37 °C in a humidified incubator with 5% CO_2_. The reagents used in this study were LPS (Cat. No. L4391; Sigma), cell counting kit-8 (CCK-8; Cat. No. NU679; Dojindo), MAO GloTM assay kit (V1402; Promega), alanine transaminase (Cat. No. C009–2–1; Nanjing Jiancheng, China), and aspartate transaminase (AST; Cat. No. C0010–2–1; Nanjing Jiancheng, China), IL-4 (KE00016; Proteintech), IL-10 (KE00170; Proteintech), and TNF-α (KE00154; Proteintech). silymarin (Cat. No. BD01452277; Bidepharm), R(−)-deprenyl (ab120604; Abcam), or recombinant human MAO-B (31503; Active Motif). All chemical reagents were purchased from commercial suppliers and used without further purification unless otherwise stated.

#### Cytotoxicity assay

HepG2 cells were seeded in 96-well plates at a density of 5 × 10^3^ cells/well in 100 μL of the culture medium and allowed to adhere overnight. The compound dissolved in dimethyl sulfoxide (DMSO) was then added to the wells at concentrations of 0, 10, 20, 40, 60, 80, and 100 μM and followed for 24-hour incubation. Cell viability was assessed using the Cell Counting Kit-8 (CCK-8) assay according to the manufacturer’s protocol. Absorbance was measured at 450 nm using a microplate reader, and cell viability was calculated as: cell viability % = [(ODm–ODb)/(ODc–ODb)]*100% (optical densities (ODs): The OD value of the measured group; ODc: The OD value of the control group; ODb: The OD value of the blank group).

#### Determination expression of ALT, AST, IL-4, IL-10, and TNF-α in LPS-induced HepG2 cells

An LPS-induced hepatocyte injury model was established in HepG2 cells by treatment with 100 ng/mL LPS, using silymarin as a positive control. After treatment with test compounds at concentrations of 10, 20, and 40 μM, the cell culture supernatant was collected and centrifuged at 1000 × g for 15 min at 4 °C. The levels of alanine aminotransferase (ALT) and aspartate aminotransferase (AST) in the supernatant were quantified using commercial assay kits according to the manufacturer’s instructions. Absorbance was measured at 510 nm using a microplate reader, and results are expressed in units per liter (U/L). Additionally, the concentrations of interleukin-4 (IL-4), interleukin-10 (IL-10), and tumor necrosis factor-α (TNF-α) in the supernatant were determined by enzyme-linked immunosorbent assay (ELISA) using corresponding commercial kits. All procedures were performed in strict accordance with the manufacturer’s protocols. Cytokine levels are reported in picograms per milliliter (pg/mL), as calculated from the respective standard curves.

### Statistical analysis

Data are presented as the mean ± standard deviation (S.D.). Student’s t-test was used for comparisons between two groups, whereas one-way analysis of variance (ANOVA) was performed for multiple group comparisons. Statistical significance was defined as *p* < 0.05.

## Results

### Isolation and structural elucidation

The enantiomers (±)-**1** were obtained as pale-yellow amorphous powders. The molecular formula was determined as C_14_H_14_O_3_ (indicating eight degrees of unsaturation), unambiguously established by a combination of NMR data (Table 1) and high-resolution ESI–MS ([M + H]^+^ at *m/z* 231.1016, calcd. 231.1016). The IR spectrum displayed characteristic absorption bands at 3415 cm^−1^ (O–H stretching vibration) and at 1654/1598 cm^−1^ (aromatic C = C skeletal vibrations), confirming the presence of hydroxyl and aromatic groups in the molecule. The ^1^H NMR spectrum (500 MHz, CD_3_OD) of **1** exhibited 13 distinct proton resonances, including: two singlets attributable to geminal methyl groups [*δ*_H_ 0.86 (3H, s) and 1.26 (3H, s)]; two aromatic singlets [*δ*_H_ 7.08 (1H, s) and 6.76 (1H, s)]; one olefinic proton [*δ*_H_ 7.50 (1H, s)]; a multiplet corresponding to an overlapped methylene group [*δ*_H_ 0.83 (2H)]; and a methine triplet [*δ*_H_ 1.73 (1H, *J* = 6.6 Hz, t)]. The ^1^³C NMR (125 MHz, CD_3_OD) and DEPT spectra revealed 14 carbon signals, classified as follows: two geminal methyl carbons (*δ*_C_ 19.7, 27.1), one carbonyl carbon (*δ*_C_ 165.3), three aliphatic carbons (*δ*_C_ 26.7, 20.4, 18.2), two olefinic carbons (*δ*_C_ 124.9, 140.6), and six aromatic carbons. All ^1^H and ^1^³C NMR signals of **1** were unambiguously assigned, as summarized in Table 1.

**Figure 2.**
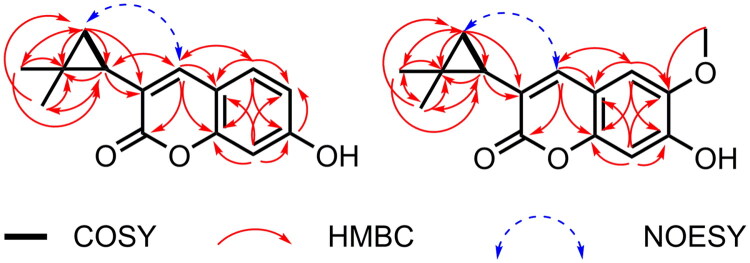
Key 1H–1H COSY, HMBC, and NOESY correlations of compounds **1** and **2**.

The ^1^H-^1^H COSY spectrum (Figure 2) revealed two distinct spin–spin coupling systems: H-1′/H-3′ and H-5/H-6. Comprehensive interpretation of the HMBC correlations enabled unambiguous assignment of proton–carbon connectivities. Specifically, long-range couplings were observed from H-4 to C-1′, C-2, C-4a, C-5, and C-8a; from H-5 to C-4, C-4a, C-7, and C-8a; from H-6 to C-4a, C-7, and C-8; and from H-8 to C-7, C-6, C-4a, and C-8a. The two methyl groups (H-4′ and H-5′) showed HMBC correlations with C-1′, C-2′, and C-3′. H-1′ exhibited cross-peaks with C-2′, C-3′, C-4′, C-5′, C-2, and C-4, while H-3′ correlated with C-1′, C-2′, C-4′, C-5′, and C-3. These HMBC data support the presence of a geminal dimethyl cyclopropane unit linked to the α-pyrone ring. Moreover, the cyclopropane ring is directly attached at the C-3 position of the benzo-α-pyrone scaffold, as evidenced by key HMBC correlations from H-3′ to C-3 and from H-1′ to C-2 and C-4. NOESY correlations further revealed a clear spatial interaction between H-4 and H-3′, whereas no such correlations were observed between H-4 and either H-4′ or H-5′ (Figure 2). These findings conclusively indicate that the geminal dimethyl cyclopropane moiety is oriented distally relative to the C-4 position within the overall molecular architecture (Figures S3–S13 for all spectrum of **1** in the supporting information).

**Figure 3.**
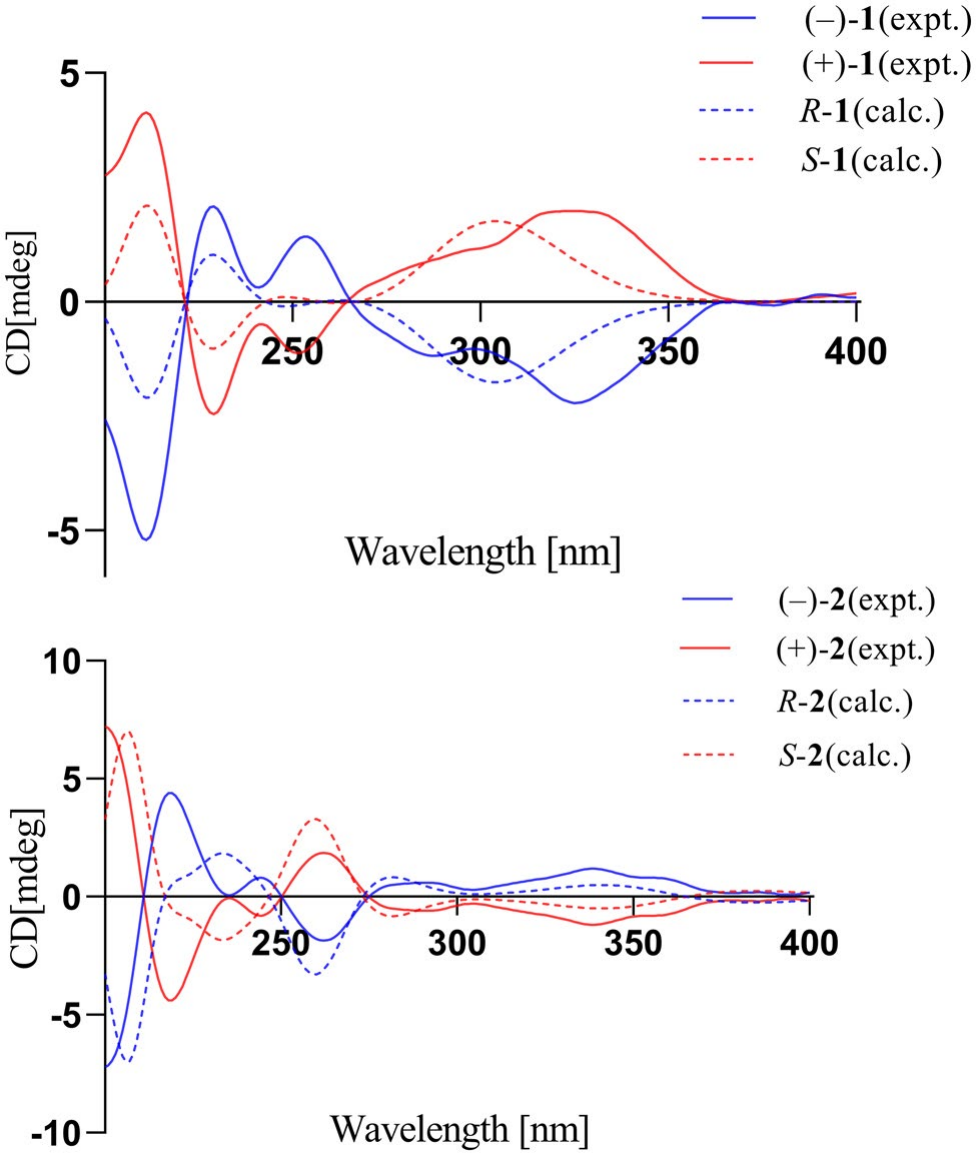
Experimental and calculated ECD spectra of (±)-**1** and (±)-**2**.

Compound **1**, The experimental circular dichroism (ECD) spectrum of compound **1** (Figure 3) exhibited no distinct Cotton effect, suggesting that it might exist as a racemic mixture. This hypothesis was confirmed by chiral high-performance liquid chromatography (HPLC), which resolved **1** into two enantiomers with an approximate 1:1 peak area ratio, indicating a racemic composition (see Figure S1 in supporting information). The absolute configurations of (+)-**1** and (−)-**1** were assigned as 1′*R* and 1′*S*, respectively, by comparing the calculated ECD spectra with experimental data.

Enantiomer 2 was obtained as a pale-yellow amorphous powder. Its molecular formula was determined to be C_15_H_16_O_4_ (eight degrees of unsaturation) based on (+)-HR-ESIMS analysis, which showed a protonated molecular ion at *m/z* 261.1125 [M + H]^+^ (calcd for C_15_H_16_O_4_, 261.1122). UV (MeOH) *λ*_max_ (log ε) was observed at 345 nm (1.78). The ^1^H NMR spectrum of **2** exhibited 15 proton resonances, including two geminal methyl singlets [*δ*_H_ 0.86 (3H, s) and 1.26 (3H, s)], two aromatic singlets [*δ*_H_ 7.08 (1H, s) and 6.76 (1H, s)], an olefinic singlet [*δ*_H_ 7.50 (1H, s)], a multiplet corresponding to an overlapped methylene group [*δ*_H_ 0.83 (2H, m)], and a methine triplet [*δ*_H_ 1.73 (1H, *J* = 6.6 Hz, t)]. The ^1^³C NMR and DEPT spectra confirmed the presence of 15 carbon signals, categorized as follows: one methoxy group (*δ*_C_ 56.8), two geminal methyl carbons (*δ*_C_ 19.7, 27.1), one carbonyl carbon (*δ*_C_ 165.6), three aliphatic carbons (*δ*_C_ 26.7, 18.2, 20.5), six aromatic carbons (*δ*_C_ 113.0, 109.5, 147.0, 151.8, 103.6, 150.0), and two olefinic carbons (*δ*_C_ 125.3, 140.7). Collectively, these spectroscopic data support the assignment of a geminal dimethyl cyclopropane unit linked to a benzo-α-pyrone scaffold at the C-3 position.

The ^1^H-^1^H COSY correlation between H-1′ and H-3′ defined one spin system. Furthermore, the benzo-α-pyrone scaffold was unambiguously established by HMBC correlations: H-4 showed long-range couplings with C-2, C-4a, C-5, C-8a, and C-1′, while H-5 correlated with C-4, C-4a, C-6, C-7, and C-8a. The methoxy group is placed at C-6 within the aromatic system, as concluded from the observed HMBC correlation of *δ*_H_ 3.90 (3H, s, H-OCH_3_) to *δ*_C_ 147.0 (C-6). These data collectively confirm the C-6 methoxylated benzo-α-pyrone core structure. The geminal dimethylcyclopropane moiety was further linked to the C-3 position of the benzo-α-pyrone core *via* a single carbon-carbon bond, as confirmed by the HMBC correlations the HMBC correlations from H-3′ to C-3 and from H-1′ to C-2 and C-4, thereby establishing the complete planar structure of compound **2.**

The geometric configurations were unambiguously determined using NOESY analysis (Figure 2). Distinct correlations were observed between H-4 (δ_H_ 7.50) and H-3′ (δ_H_ 1.73), but no interactions were detected between H-4 and either H-4′ (δ_H_ 0.86) or H-5′ (δ_H_ 1.26). These NOESY data conclusively position the geminal dimethylcyclopropane moiety distal to the C-4 center within the molecular architecture of compound **2**. The experimental circular dichroism (CD) spectrum (Figure 3) displayed no detectable Cotton effects, which is consistent with the isolation of a racemic mixture. Chiral HPLC analysis confirmed this, resolving compound **2** into (+)-**2** and (−)-**2** with two peaks showing an approximate 1:1 area ratio (Figure S2 in supporting information). Therefore, the absolute configurations of (+)-**2** and (–)-**2** were assigned as 1′*R* and 1′*S*, respectively, by comparing the calculated ECD spectra with experimental data (Figures S17–S26 for all spectrum of **2** in the supporting information).

### Chemical synthesis of Rutacycoumarin A (1) and B (2)

Direct C3 alkylation of readily available coumarins with alkyl radicals through radical substitution is an attractive synthetic strategy that provides a straightforward and versatile route to diverse derivatives of these compounds (Bloem et al. 2021; Özay 2025). N-hydroxyphthalimide esters are widely used as precursors for generating alkyl radicals because of their high efficiency. However, there are few reports on C3 alkylation methods for coumarins with substituents on the phenyl ring (Lam et al. 2016; Stefanachi et al. 2018; Datta et al. 2023; Zhu et al. 2023; He et al. 2024), and there are no reports on direct C3 alkylation of hydroxy-substituted coumarins. This poses a challenge for the synthesis of compounds **1** and **2.**

The syntheses of **1** and **2** are presented in Table 2. Initially, we followed literature conditions; however, no target products were obtained under the reported reaction conditions (Table 2, Entries **1–5**). Consequently, we conducted a series of investigations into the direct C3 alkylation of this hydroxy-substituted coumarin. We found that adding the catalyst DIPEA to the reaction from Entry **2** resulted in isolation compound **1** with a yield of 15%. Changing the LED lamp from 455 to 365 nm slightly improved the yield (Table 2, Entries **6–7**). The direct reaction of N-hydroxyphthalimide ester **4** with umbelliferone significantly enhanced its yield. Further removal of the inorganic base Li_2_CO_3_ under the co-catalysis of 10 mol% DIPEA and Na_2_S successfully afforded compound **1** with a yield of 33% (Table 2, Entries **8–9**). Removing either the catalyst or replacing the irradiation source with white light caused a marked decrease in the yield (Table 2, Entries **10–12**). The substitution of Na_2_S with other sulfur species as electron donors, including Cu_2_S, CuS, Bi_2_S_3_, and PhSNa, resulted in decreased reaction yields (Table 2, Entries **13–16**). Given that Potassium ethyl xanthate and *N*-hydroxyphthalimide esters **4** may form photoactive electron donor-acceptor (EDA) complexes (Song et al. 2023) that generate 2,2-dimethoxycyclopropyl radicals under light irradiation for the reaction, replacing Na2S with 10 mol% potassium ethyl xanthate ultimately facilitated the synthesis of natural product 1 in 35% yield *via* a direct C3 alkyl radical substitution reaction (Table 2, entry **17**). Under these reaction conditions, using commercially available scopoletin as a starting material, compound **2** was successfully synthesized with a yield of 26%. The synthetic spectral data of compounds **1** and **2** were consistent with the separated data, further validating the structures of these natural products (Song et al. 2023).

**Table 2.** Synthesis of compounds **1** and **2**.

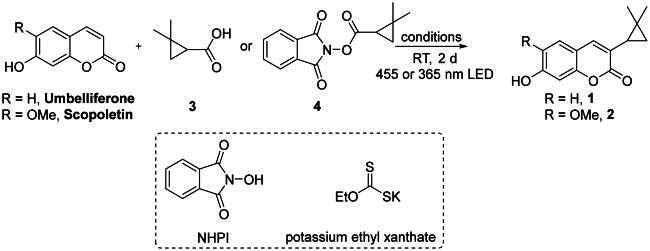		
Entry	Conditions*^a^*	Yield of 1^b^
1 (Song et al. 2023)	**3**, NHPI, DIC, Na_2_S, 455 nm LED, DMF, RT, 2 d	N.D.
2 (Zhou et al., 2022)	**3**, NHPI, DIC, DIPEA, 455 nm LED, Li_2_CO_3_, DMSO, RT, 2 d	N.D.
3 (Liu et al. 2019)	1. **3**, NHPI, DCC, DMAP, CH_2_Cl_2_, RT, 3h; 2. Ru(bpy)_3_Cl_2_·6H_2_O, DABCO, DMA, rt, 450 nm LED, overnight	N.D.
4 (Jafarpour et al. 2019)	**4**, K_2_S_2_O_8_, K_2_CO_3_ (2.0 equiv), ACN/H_2_O (1/5), 120 °C,16 h	N.D.
5 (Gan et al. 2022)	**4**, PPh_3_, NaI, TMEDA, DMF, 450 nm LED, 3h	N.D.
6	**3** (2.0 eq), NHPI (2.0 eq), DIC(2.0 eq), Na_2_S (20 mol%), DIPEA (20 mol%), Li_2_CO_3_ (1.2 eq), 455 nm LED, DMSO, RT, 2 d	15%
7	**3** (2.0 eq), NHPI (2.0 eq), DIC(2.0 eq), Na_2_S (20 mol%), DIPEA (20 mol%), Li_2_CO_3_ (1.2 eq), 365 nm LED, DMSO, RT, 2 d	18%
8	**4** (1.0 eq), DIPEA (10 mol%), Na_2_S (10 mol%), Li_2_CO_3_ (1.2 eq), 365 nm LED, DMSO, RT, 2 d	29%
**9**	**4 (1.0 eq), DIPEA (10 mol%), Na_2_S (10 mol%), 365 nm LED, DMSO, RT, 2 d**	**33%**
10	**4** (1.0 eq), Na_2_S(10 mol%), 365 nm LED, DMSO, RT, 2 d	trace
11	**4** (1.0 eq), Na_2_S(10 mol%), Li_2_CO_3_(1.2 equiv), 365 nm LED, DMSO, RT, 2 d	26%
12	**4** (1.0 eq), DIPEA(10 mol%), Na_2_S(10 mol%), white LED, 3 d	N.D.
13	**4** (1.0 eq), DIPEA(10 mol%), Cu_2_S(10 mol%), 365 nm LED, DMSO, RT, 2 d	18%
14	**4** (1.0 eq), DIPEA(10 mol%), CuS(10 mol%), 365 nm LED, DMSO, RT, 2 d	16%
15	**4** (1.0 eq), DIPEA(10 mol%), Bi_2_S_3_(10 mol%), 365 nm LED, DMSO, RT, 2 d	7%
16	**4** (1.0 eq), DIPEA(10 mol%), PhSNa(10 mol%), 365 nm LED, DMSO, RT, 2 d	11%
**17**	**4 (1.0 eq), DIPEA(10 mol%), potassium ethyl xanthate (10 mol%), 365 nm LED, DMSO, RT, 2 d**	**35%**
18	**4** (1.0 eq), potassium ethyl xanthate (10 mol%), 365 nm LED, DMSO, RT, 2 d	15%

### The recombinant human MAO-B enzyme inhibitory activities of the four compounds

Subsequently, four new compounds isolated from the *R. graveolens* extracts were assayed for MAO-B inhibition. Our results found that compounds (+)-**1** and (−)-**1** inhibited MAO-B with IC_50_ values of 7.26 μM and 8.72 μM, respectively (Table 3). These results indicate that compounds (+)-**1** and (−)-**1** have significant MAO-B inhibitory activity. In contrast, (+)-2 and (−)-2 exhibited moderate inhibition with IC_50_ values of 22.1 μM and 30 μM.

**Table 3.** Inhibition of recombinant human MAO-B by compounds isolated from *Ruta graveolens* L. (*n* = 3).

Compounds	Residual activity at 10 μM (%)	IC_50_ (μM)
(+)-**1**	61	7.26
(−)-**1**	64	8.79
(+)-**2**	37	22.1
(−)-**2**	37	30
R(-)-deprenyl	ND	0.053

### Cytotoxicity screening

**Figure 4.**
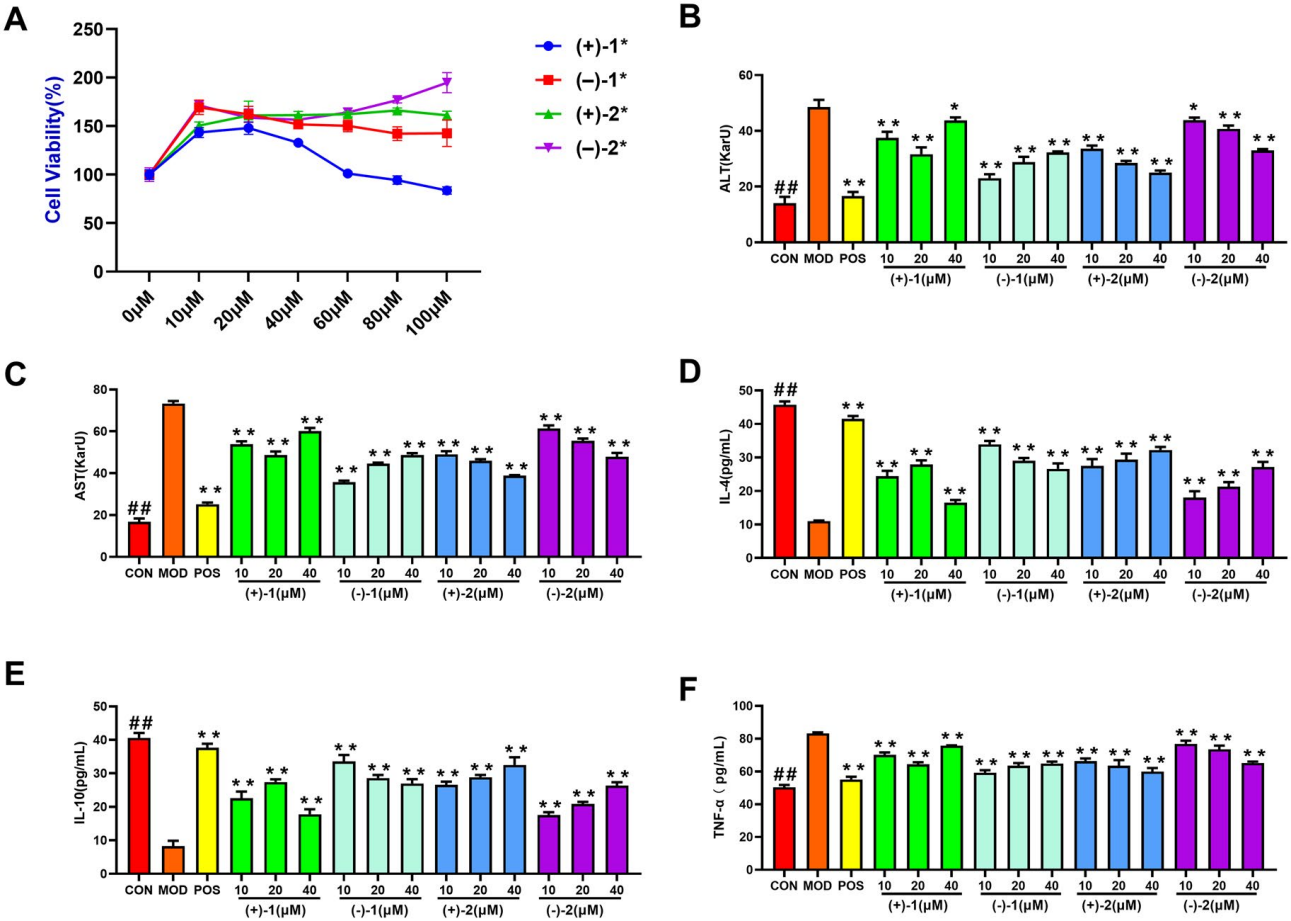
Compounds attenuate lipopolysaccharide (LPS)-induced cellular damage and inflammatory response in HepG2 cells. (A) Cell viability of HepG2 cells treated with the compound at concentrations of 0, 10, 20, 40, 60, 80, or 100 μM was measured by CCK-8 assay. (B) ALT levels in cell supernatants. (C) AST levels in cell supernatants. (D) IL-4 levels in supernatants. (E) Apigenin reduces IL-10 levels. (F) Apigenin inhibits TNF-α production. Data are mean ± SD (*n* = 3); *p < 0.05, **p < 0.01 vs.MOD. ##*p* < 0.01 vs.MOD. Groups: CON (untreated control), MOD (LPS-treated), POS (silibinin, 50 μM).

To evaluate the cytotoxic effects of the chiral compounds on HepG2 cells, we systematically assessed (+)-**1**, (−)-**1**, (+)-**2**, and (−)-**2** at concentrations ranging from 10 to 100 μM over 24 h using the CCK-8 assay (Figure 4A). (+)-**1** exhibited dose-dependent cytotoxicity above 80 μM (*p* < 0.05, vs. vehicle control), whereas (−)-**1**, (+)-**2**, and (−)-**2** showed no significant viability reduction (≥ 98% survival, *p* > 0.05) at any tested doses.

### The effects of four compounds on the ALT, AST, TNF-α, IL-4 and IL-10 levels of LPS-induced HepG2 cells

An LPS-induced hepatocyte injury model (100 ng/ml) was established using HepG2 cells. Given these toxicity profiles and high compound isolation yields (> 85%), we selected 10 μM (low), 20 μM (medium), and 40 μM (high) as nontoxic working concentrations for subsequent mechanistic studies, ensure biological relevance in downstream analyses. ALT and AST levels, well-established biomarkers of hepatic damage, were quantified in the cell supernatants to assess compound efficacy. Compounds (+)-**1**, (−)-**1**, and (+)-**2** significantly reduced LPS-elevated ALT and AST levels in HepG2 cells (*p* < 0.01 vs. model). Dose-dependent suppression was most pronounced for (+)-**2** and (−)-**2**, whereas (−)-**1** showed maximum efficacy at 10 μM (Figure 4B,C).

LPS triggers robust inflammation through the overexpression of the proinflammatory cytokines. We evaluated compound efficacy by quantifying TNF-α (pro-inflammatory), IL-4, and IL-10 (anti-inflammatory) levels in HepG2 supernatants using ELISA. Compared with the controls, LPS significantly increased TNF-α (*p* < 0.01) and decreased IL-4 and IL-10 levels, confirming inflammation. Treatment with (±)-**2** (10–40 μM) dose-dependently restored IL-4 and IL-10 levels (*p* < 0.01) and suppressed TNF-α expression, with (−)-**1** showing exceptional potency at 10 μM (Figure 4D–F). These results demonstrated that compounds (+)-**1**, (−)-**1**, and (+)-**2** significantly inhibited LPS-induced inflammatory response.

## Discussion

Natural products represent a rich source of chemical diversity and have historically provided unique molecular scaffolds for drug discovery, as exemplified by artemisinin, taxol, and penicillin. The recently identified compounds (±)-Rutacycoumarin A (**1**) and (±)-Rutacycoumarin B (**2**) feature unprecedented benzofuran scaffolds incorporating cyclopropane motifs, offering a novel molecular template for future drug development. While no coumarin isomers other than the enantiomers we previously reported have been found in *R. graveolens* (Liu et al. 2023*)*, further investigation into the coumarin constituents of this species should address the occurrence and biological significance of their enantiomeric forms.

The generally low abundance of natural products in their sources often limits bioactivity evaluation, making chemical synthesis a crucial tool for enabling such studies. However, the structural complexity and multiple chiral centers of many natural products pose considerable synthetic challenges. In contrast, (±)-Rutacycoumarins A (**1**) and B (**2**) possess only a single chiral center, which allowed their total synthesis to be efficiently achieved in a one-step reaction. Although no stereoselective control was applied in this process—resulting in nearly racemic mixtures (enantiomeric ratio ≈ 1:1)—the simplified synthetic access to these scaffolds paves the way for future efforts in asymmetric synthesis and further pharmacological exploration.

**Figure 5.**
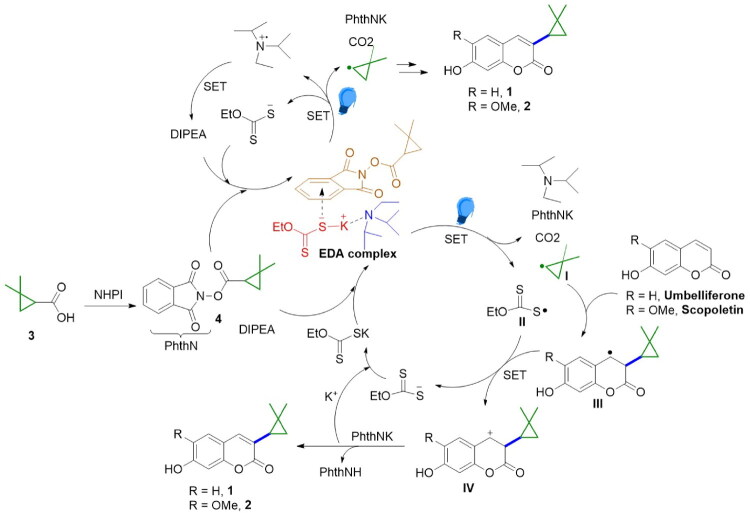
Possible reaction mechanism.

This paper presents a photoinduced C3-alkylation approach catalyzed by DIPEA and potassium ethyl xanthate. Building on prior screening and relevant literature (de Pedro Beato et al. 2021; Zhou et al., 2022; Song et al. 2023), we propose a plausible mechanism involving electron-donor–acceptor (EDA) interactions (Figure 5). An EDA complex forms among the NHPI ester **4**, DIPEA, and potassium ethyl xanthate. Upon light irradiation, this complex undergoes a single-electron transfer, generating an alkyl radical, a sulfur radical **II**, a phthalimide salt (PhthNK), and CO2. The alkyl radical adds to the C3 position of umbelliferone or scopoletin, yielding a radical intermediate **III**. This intermediate is oxidized by the sulfur radical **II** to give a carbocation intermediate **IV**, which is subsequently generated *via* another SET event. Deprotonation of **IV**, assisted by PhthNK, affords the target products (**1** or **2**), while the xanthate catalyst is reconstituted to close the catalytic cycle. Meanwhile, DIPEA can also act as an electron donor to form EDA complexes that generate alkyl radicals *via* SET upon light irradiation, driving the C3-alkylation. It is worth mentioning that the above-proposed EDA complexes still require further experimental confirmation *via* UV/Vis spectroscopic analysis.

*R. graveolens* exhibits a range of pharmacological activities, with antibacterial and anti-inflammatory effects being the most frequently reported (Luo et al. 2024). Coumarins derived from this plant have demonstrated multiple bioactivities, including antibacterial, anti-inflammatory, anticancer/antiproliferative, acetylcholinesterase (AChE) inhibitory, and monoamine oxidase B (MAO-B) inhibitory properties (Luo et al. 2024). Monoamine oxidases (MAOs), which exist as isoforms A and B, are key targets in Parkinson’s disease due to their role in neurotransmitter degradation and association with neurological disorders. In inhibitory assays, the dichloromethane extract of *R. graveolens* (9.78 mg/mL) and its isolated compound rutamarin (6.17 μM) strongly inhibited human MAO-B, with inhibition rates of 89.98% and 95.26%, respectively. Although the extract also inhibited MAO-A by 88.22%, rutamarin exhibited significantly greater selectivity for MAO-B, showing only 25.15% inhibition of MAO-A (Kozioł et al. 2020). Umbelliferone derivatives demonstrated varying levels of inhibitory activity against MAO, as reported in (Dhiman et al. 2018). Notably, the compound 2-oxo-2H-chromen-7-yl 2-hydroxy-2-phenylacetate showed significant inhibition of human MAO-B (hMAO-B), with an IC_50_ value of 10.32 ± 0.044 µM and a notably high selectivity index of 8.55. The introduction of the 2-hydroxy-2-phenylacetate moiety onto the 2-oxo-2H-chromene scaffold was found to facilitate key binding interactions within the active site of hMAO, contributing to its enhanced inhibitory potency (Dhiman et al. 2018). Evaluation of six umbelliferone derivatives, umbelliferone, 6-formyl umbelliferone, 8-formyl umbelliferone, umbelliferone-6-carboxylic acid, esculetin, and scopoletin, revealed a consistent selectivity for hMAO-A versus hMAO-B. Among them, 6-formyl umbelliferone was the most potent hMAO inhibitor (IC_50_ = 3.23 µM for hMAO-A; 15.31 µM for hMAO-B). Enzyme kinetic studies further established that both 6-formyl umbelliferone and 8-formyl umbelliferone function as competitive hMAO inhibitors (Seong et al. 2019). Differences in antioxidant or MAO-B inhibitory effects among *R. graveolens*-derived compounds might also arise from variations in phenolic composition or substitution patterns, as observed in other medicinal plants such as *M. charantia* and *D. stramonium* (Alper and Ozay 2022).

Although less potent than the reference inhibitors clorgyline and selegiline (which showed >99% inhibition at ∼3–5 μM), both the extract and rutamarin exhibited considerable MAO inhibitory activity, suggesting their potential as candidates for Parkinson’s disease drug development. In this study, we demonstrated that coumarins from *R. graveolens*—particularly those bearing substituents at the C-3 position—display potent MAO-B inhibitory activity. Moreover, the absolute configuration at the C-1′ position in (±)-Rutacycoumarins A (**1**) and B (**2**) exerted no significant influence on MAO-B inhibition. However, the MAO-A/B selectivity of these four compounds remains to be investigated, and their activities have not yet been validated in cellular or *via* models. Although hepatoprotective effects have been previously reported against graveoline- a phenylquinoline alkaloid from *R. graveolens*- no such activity has been documented for its coumarin derivatives (Schelz et al. 2016). In the present study, all four tested compounds exhibited markedly reduced efficacy compared to the positive controls, and their hepatoprotective potential lacks support from *in vivo* or mechanistic studies.

## Conclusions

In conclusion, two enantiomeric pairs, (±)-Rutacycoumarin A (**1**) and (±)-Rutacycoumarin B (**2**), exhibiting identical structural frameworks but distinct stereochemical configurations, were isolated from an aqueous extract of *R. graveolens*. The enantiomers were resolved *via* chiral-phase HPLC, with absolute configurations unambiguously assigned through comparative analysis of electronic circular dichroism (ECD) spectra, optical rotation data, and corroborative spectroscopic evidence. A novel photoactivation system was employed to synthesize (±)-Rutacycoumarin A (**1**) and (±)-Rutacycoumarin B (**2**), using DIPEA and potassium ethyl xanthate as catalytic electron donors. The utilization of C3 direct alkylation of coumarin enables efficient synthesis of diverse alkylated coumarin derivatives, accelerating structure–activity relationship (SAR) studies and facilitating the identification of potential drug candidates with enhanced biological activity and druggability. These enantiomeric coumarins demonstrate stereospecific anti-inflammatory effects in LPS-stimulated HepG2 cells by differentially modulating IL-4, IL-10, and TNF-α expression, while concurrently attenuating ALT and AST elevation. Additionally, all four enantiomeric coumarins displayed inhibitory activity against MAO-B. The study of enantiomers provides new insights into the biological mechanisms and synthetic strategies of coumarin derivatives.

## Supplementary Material

Supplementary information.docx

## Data Availability

Data will be made available on reasonable request from Yueping Jiang.
