# Supplementary material for: (±)-Rutacycoumarins A and B: two pairs of unprecedented coumarin enantiomers from the aerial part of Ruta graveolens L. and chemically synthesized
Source: Pharm Biol. 2025 Dec 13;64(1):1–16. doi: 10.1080/13880209.2025.2600291 (PMC12704139; doi:10.1080/13880209.2025.2600291)
Supplement: Supplementary information.docx [file IPHB_A_2600291_SM1176.docx]

**Supplementary data**

**(±)-**Rutacycoumarins A and B: two pairs of unprecedented coumarin enantiomers from the aerial part of *Ruta graveolens* L. and chemically synthesized

Xu Feng^a,c,§^, Jing Fang^a,c, §^, Yanyang Liu^a,c^, Chengyu Ge^a,c^, Xiaolin Liao^d^, Tao Jiang^e^, Xiongjun Hou ^f^, Hao Huang^g^, Shao Liu^a,c,^^h^, Aimin Wang ^b,c,e,^[[1]](#footnote-1)^*^, Yueping Jiang^a,c,d, ^[[2]](#footnote-2)^*^

^a^ Department of Pharmacy, Xiangya Hospital, Central South University, Changsha 410008, Hunan, China

^b^ Department of Emergency, Xiangya Hospital, Central South University, Changsha 410008, Hunan, China.

^c^ National Clinical Research Center for Geriatric Disorders, Xiangya Hospital, Central South University, Changsha 410008, China

^d^ Department of Clinical Pharmacy, Hunan University of Medicine General Hospital, Huaihua, 418000, Hunan, China

^e^ Department of Emergency, Hunan University of Medicine General Hospital, Huaihua 418000, Hunan, China.

^f^ Department of Pharmacy, Jiangxi Provincial People’s Hospital, the First Affiliated Hospital of Nanchang Medical College, Nanchang 330006, China

^g^Jiangxi Province Key Laboratory of Pharmacology of Traditional Chinese Medicine, School of Pharmacy, Gannan Medical University, Ganzhou 341000, China.

^h^State Key Laboratory Neurology and Oncology Drug Development, Nanjing 210018, China.

^§^ These authors contributed equally to this work

*Corresponding author

1. mail: [wangaimin@csu.edu.cn](mailto:wangaimin@csu.edu.cn) (Aimin Wang); [jiangyueping@csu.edu.cn](mailto:jiangyueping@csu.edu.cn) (Yueping Jiang)

**Tables of contents**

[1. General comment ……………………………………………………………………………………………………………S1](#_Toc199514989)

[2. Extraction and isolation of (±)1 and (±)2…………………………………….…………………………………S2](#_Toc199514990)

[2.1 Plant material ……………………………………………………………………………………………………………S2](#_Toc199514991)

[2.2 Extraction and isolation ……………………………………………………………………………………………S2](#_Toc199514992)

[2.3 Spectroscopic data ………………………………………………………………………………………………………S2](#_Toc199514993)

[3. ECD calculation ………………………………………………………………………………………………………………S3](#_Toc199514994)

[4. Synthesis of compound 1 and 2 …………………………………………………………………………………………S3](#_Toc199514995)

[5. Biological activity assay ……………………………………………………………………………………………………S5](#_Toc199514996)

[6. Experimental details, ^1^H, ^13^C NMR, HRMS and HPLC spectra ………………………………………S6](#_Toc199514997)

# General comment

Optical rotations were determined using an INESA SGW-3 automatic polarimeter fitted with a 10-mm micro-polarizer. UV spectra were acquired by a Cary 300 UV - Vis spectrophotometer. Circular dichroism (CD) spectra were determined using a JASCO J-815 spectrometer, while infrared (IR) spectra were recorded with a Nicolet IS50 Fourier-transform infrared spectrometer (Thermo Fisher Scientific Co. Ltd., China).

One-dimensional and two-dimensional nuclear magnetic resonance (NMR) spectra were measured in CD_3_OD solvent using Bruker NMR spectrometers (Bruker AVANCE III 400 M and Bruker AVANCE NEO 600 M, Bruker, Ltd, Germany). The magnetic field strengths were 9.4 T (for the 500-M instrument) and 14.1 T (for the 600-M instrument), respectively. The peak of tetramethylsilane (TMS) was used as the reference standard for chemical shift.

High-resolution electrospray ionization mass spectrometry (HR - ESI - MS) data were obtained by coupling an Agilent 1290 HPLC instrument (equipped with a 1290 Flexible Pump and an Agilent G7114B DAD absorbance detector, Agilent Technologies, Ltd., USA) with an accurate - mass Q - TOF (G6545B, Agilent Technologies, Ltd., USA). A C18 column (2.1 × 100 mm, 1.8-μm, Agilent Technologies, Ltd., USA) was employed for separation. The gradient elution program was optimized per sample to ensure efficient chromatographic separation and accurate compound detection. High - performance liquid chromatography (HPLC) separation was performed on an instrument equipped with an Agilent ChemStation for the LC system, a G1311A Quat Pump, and an Agilent G1315B DAD absorbance detector (Agilent Technologies, Ltd., USA), using a YMC - Pack ODS - A (250 mm × 10 mm I.D., YMC Co. Ltd, Japan) semi - preparative column. HPLC chiral separation was carried out on an instrument equipped with an Agilent ChemStation for the LC system, a 1260 Quit Pump VL, an Agilent G7114A VWD absorbance detector, and a G1364F fraction collector (Agilent Technologies, Ltd., USA), using a CHIRALPAK AD - H column (0.46 cm I.D. × 25 cm, Daicel Chiral Technologies Co. Ltd., China).

Column chromatography (CC) was performed using microporous adsorbent resin D101 (0.3 - 1.25 mm, Weibo Technology CO., Ltd, Guangdong, China) and silica gel (200 - 300 mesh, Qingdao Marine Chemical Inc., China). The elution progress was monitored by thin-layer chromatography (TLC) to collect the target components promptly. Flash chromatography was performed using Sephadex LH-20 (Pharmacia Biotech AB, Sweden) following the standard operating procedures. TLC was used to monitor the eluate in real-time to ensure the effective separation of the target compounds.

TLC was conducted on pre-coated silica gel GF254 plates. The developing solvent was selected according to the properties of the samples. Spots were visualized by exposure to ultraviolet light (254 or 301 nm) or by spraying with a solution of H_2_SO_4_–H_2_O (1:9) in EtOH (9:1) followed by heating. All chemical reagents were purchased from commercial suppliers and, unless otherwise stated, were used without further purification. All solvents used for chromatography, UV, IR, CD, and MS analyses were of Lab-Scan HPLC grade and were filtered through 0.45-μm and 0.22-μm membrane filters (Keyilong Lab Equipment Co., Ltd, Tianjin, China) to remove impurities and ensure the accuracy of the experimental results.

# Extraction and isolation of (±)1 and (±)2

## 2.1 Plant material

Dried aerial parts of R. graveolens were collected from Guangning County (23°37'N, 112°20'E; Zhaoqing City, Guangdong Province, China) for experimental analysis. Botanical authentication was performed by Prof. Shao Liu of Xiangya Hospital, Central South University, with voucher specimens (ID 2,020,001) archived in the Medicinal Chemistry Laboratory at Xiangya Hospital's Pharmacy Building for long-term preservation and future. The authenticated voucher specimen (RG-ZQ2020-01) has been permanently catalogued in the authors’ laboratory, located at 87 Xiangya Road, Changsha 410008, China.

## 2.2 Extraction and isolation

Aerial parts of dried R. graveolens (10 kg) were mechanically ground and subjected to dual-phase ultrasonic-assisted extraction (8 L ultrapure water × 1 h per cycle, 25°C) using a Branson 5800 system. Combined filtrates were vacuum-concentrated (Rotavapor R-300, Büchi; 40°C, 15 mbar) to yield an aqueous extract (1,823.7 g). Primary fractionation employed D101 macroporous resin with sequential elution: 5 L H2O(A), 4 L 50% EtOH (B), and 5 L 90% EtOH (C; 2). Visual elution termination criteria were applied when effluent absorbance dropped below 0.05 AU. The ethanol-rich fraction (C) was lyophilized to obtain 14.3 g of dark-brown crude extract (0.78% recovery rate). Following solvent removal, fraction C (14.3 g) was subjected to silica gel chromatography using a methanol (0% to 100%) gradient in dichloromethane. The resulting subfractions YXC-1 to YXC-11 were identified by TLC. YXC-3 was further purified by Sephadex LH-20 column chromatography using a solvent system of petroleum ether: dichloromethane: methanol (5:5:1). The resulting fractions were identified by TLC and combined to yield 10 fractions (YXC-3-1 to YXC-3-10). YXC-3-7 was then purified using a thin-layer preparative plate with a solvent system of petroleum ether: ethyl acetate (4:1), resulting in three fractions (YXC-3-7-1 to YXC-3-7-3). YXC-3-7-5 was further purified using semi-preparative HPLC (Ph column, ACN-H_2_O-0.1% FA, v/v, 38:62, 2.0 ml/min), yielding compound **1** (4.6 mg, *t*_R_ = 34.0 min). YXC-3-7-2 was further purified using semi-preparative HPLC (Ph column, ACN-H_2_O-0.1% FA, v/v, 38:62, 2.0 ml/min), yielding compound **2** (4.3 mg, *t*_R_ = 32.3 min). Subsequent separation of **1** by semipreparative HPLC using a normal phase chiral column (CHIRALPAK AD-H) and eluting with n-hexane/isopropyl alcohol (9:1, 0.7 ml/min) afforded compound (–)-**1** (2.0 mg, *t*_R_ = 10.721 min) and compound (+)-**1** (2.1 mg, *t*_R_ = 19.197 min), respectively. Similarly, separation of **2** by HPLC using a normal phase chiral column (CHIRALPAK AD-H) and eluting with n-hexane/isopropyl alcohol (9:1, 0.7 ml/min) obtained compound (+)-**2** (1.6 mg, *t*_R_ = 12.385 min) and compound (–)-**2** (1.7 mg, *t*_R_ = 16.028 min), respectively.

## 2.3 Spectroscopic data

(1ʹ*S*-**1**): white amorphous powder, [α]₂₅ᴰ +103.8 (*ⅽ* 0.025, MeOH); UV (MeOH) λₘₐₓ (log *ε*) = 330 (1.81) nm; CD (MeOH) λₘₐₓ = 211 (mdeg –5.298), 229 (mdeg +2.178), 254 (mdeg +1.438), 32 (mdeg –2.251) nm; IR (KBr) νₘₐₓ 3416, 3285, 2925, 2854, 1599, 1260, 1192, 1120, 862 cm⁻¹;¹H NMR (CD_3_OD, 500 MHz) and ¹³C NMR (CD_3_OD, 125 MHz) spectroscopic data, see Table 1, HR-ESIMS *m/z* 231.1016 [M + H]⁺ (calcd for C₁₄H₁₅O₃, 231.1016).

(1ʹ*R*-**1**): white amorphous powder, [α]₂₅ᴰ –90.0 (ⅽ 0.036, MeOH); The UV and IR spectra of (–)-**1** exhibited characteristic absorptions similar to those of (+)-**1**. CD (MeOH) λₘₐₓ = 211 (mdeg +4.251), 228 (mdeg –2.538), 252 (mdeg –1.176), 328 (mdeg +1.985) nm;¹H-NMR (CD_3_OD, 500 MHz) and ¹³C-NMR (CD_3_OD, 125 MHz) spectroscopic data, see Table 1, HR-ESIMS *m/z* 231.1016 [M + H]⁺ (calcd for C₁₄H₁₅O₃, 231.1016).

(2ʹ*S*-**2**): faint yellow amorphous powder, [α]₂₅ᴰ +60.5 (ⅽ 0.096, MeOH); UV (MeOH) λₘₐₓ (log *ε*) = 345 (1.78) nm; CD (MeOH) λₘₐₓ = 219 (mdeg –4.391), 244 (mdeg –0.812), 262 (mdeg +1.861), 339 (mdeg –1.190) nm; IR (KBr) νₘₐₓ 2922.3, 2851.5, 1709.5, 1583.0, 1274.8, 1149.9, 1036.2 cm⁻¹; NMR (CD_3_OD, 600 MHz) and ¹³C-NMR (CD_3_OD, 150 MHz) spectroscopic data, see Table 1, HR-ESIMS *m/z* 261.1125 [M + H]⁺ (calcd for C₁₅H₁₇O₄, 261.1122).

(2ʹ*R*-**2** ) faint yellow amorphous powder, [α]₂₅ᴰ –51.0 (*ⅽ* 0.094, MeOH); The UV and IR spectra of (–)-**2** exhibited characteristic absorptions similar to those of (+)-**2**. CD (MeOH) λₘₐₓ = 218 (mdge +4.496), 244 (mdeg +0.875), 263 (mdeg –1.866), 338 (mdeg +1.206) nm;¹H-NMR (CD_3_OD, 600 MHz) and ¹³C-NMR (CD_3_OD, 150 MHz) spectroscopic data, see **Table 1**, HR-ESIMS *m/z* 261.1125 [M + H]⁺ (calcd for C₁₅H₁₇O₄, 261.1122).

# ECD calculation

We employed the time-dependent density functional theory (TDDFT) at the mPW1PW91/6 - 311g(d) level, incorporating the IEF-PCM solvent model for methanol (MeOH), to compute its theoretical electronic circular dichroism (ECD). A conformational search for each diastereomer was executed using the MMFF94 force field to initiate the process. Subsequently, the software Crest was utilized to explore the conformers of compound 1 at the GFNFF level of theory. Following the search, these conformers were optimized at the GFN2-XTB level, with a 4 kcal/mol energy window applied to eliminate high-energy conformers. In the ECD calculation, 20 excited states were considered for each conformer. The generation of Boltzmann-averaged ECD spectra was achieved through the SpecDis v1.71 software. A sigma/gamma value of 0.35 eV was set for this process, with the calculations based on the Gibbs free energy of the conformers. Boltzmann-averaged ECD spectra were achieved through the SpecDis v1.71 software (as described by Bruhn et al. in 2017). A sigma/gamma value of 0.35 eV was set for this process, with the calculations based on the conformers' Gibbs free energy.

# Synthesis of compound 1 and 2

**Synthesis of compound 4**

The 2,2-dimethylcyclopropanecarboxylic acid (1 g, 8.76 mmol), N-hydroxyphthalimide (1.57 g, 9.63 mmol), 4-dimethylaminopyridine (DMAP, 107.0 mg, 0.88 mmol), N, N'-diisopropylcarbodiimide (DIC, 1.7 g, 9.63 mmol), and dichloromethane (8.8 ml) were added in an oven-dried flask with a magnetic stirring bar. The reaction mixture was stirred at room temperature for 3 hours until the reaction was complete. Concentrate under reduced pressure to remove dichloromethane, and the residue was purified by recrystallization (diethyl ether) to afford 4. 4 was used without further purification.

**Typical Procedure for the Synthesis of Compound 1 and 2**

To a 10 mL Schlenk flask equipped with a magnetic stirring bar were added umbelliferone (1.0 mmol, 160 mg), 4 (1.0 mmol, 260 mg), Na_2_S (13 mg, 0.1 mmol), potassium ethyl xanthate (16 mg, 0.1 mmol) and DMSO (2.5 mL). The resulting mixture was charged with N_2_ and irradiated by 365 nm LEDs (36 W) for 48 h. The reaction mixture was extracted with dichloromethane. The organic layer was dried over Na_2_SO_4_, filtered, and concentrated under reduced pressure. The residue was purified by silica gel flash chromatography to give the corresponding product 1 (81 mg, 35%). ^1^H NMR (500 MHz, CD_3_OD) *δ*_H_ 7.48 (s, 1H), 7.40 (d, *J* = 8.5 Hz, 1H), 6.77 (dd, *J* = 8.5, 2.3 Hz, 1H), 6.71 (d, *J* = 2.1 Hz, 1H), 1.73 (t, *J* = 7.1 Hz, 1H), 1.27 (s, 3H), 0.87 (s, 3H), 0.81 (dt, *J* = 8.0, 5.1 Hz, 2H). ^13^C NMR (100 MHz, CD_3_OD) (*δ*_C_ 165.3, 162.0, 155.8, 140.6, 129.9, 124.9, 114.3, 113.5, 103.0, 27.0, 26.6, 20.4, 19.7, 18.1). These data are consistent with the separated spectral data.

To a 10 mL Schlenk flask equipped with a magnetic stirring bar were added scopoletin (0.1 mmol, 20 mg), 4 (0.1 mmol, 26 mg), Na_2_S (1.3mg, 0.01 mmol), potassium ethyl xanthate (1.6 mg, 0.01 mmol), and DMSO (0.25 mL). The resulting mixture was charged with N_2_ and irradiated by 365 nm LEDs (36 W) for 48 h. The reaction mixture was extracted with dichloromethane. The organic layer was dried over Na_2_SO_4_, filtered, and concentrated under reduced pressure. The residue was purified by silica gel flash chromatography to give the corresponding product 2 (6.8 mg, 26%). ^1^H NMR (400 MHz, CD_3_OD) [*δ*_H_ 7.50 (1H,s), 7.08 (1H,s), 6.77 (1H,s), 3.91 (1H,s), 1.74 (1H, *J* = 7.6, 6.2, 1.0 Hz, ddd), 1.27 (3H,s), 0.87 (3H,s), 0.81 (2H, *J* = 8.1, 5.0 Hz, dt)]. ^13^C NMR (100 MHz, CD_3_OD) *δ*_C_ (165.6, 151.7, 149.9, 147.0, 140.7, 125.2, 112.9, 109.5, 103.6, 56.8, 27.0, 26.7, 20.4, 19.7, 18.2). These data are consistent with the separated spectral data.

# Biological activity assay

HepG2 cells were acquired from the American Type Culture Collection (ATCC) and cultured in Minimum Essential Medium (MEM) supplemented with 10% fetal bovine serum (FBS), 100 U/mL penicillin, and 100 μg/mL streptomycin. Cells were maintained at 37°C in a humidified incubator with 5% CO₂. HepG2 cells were seeded into 96-well plates at a density of 5×10^3^ cells/well in 100 μL of culture medium and allowed to adhere overnight. The compound dissolved in dimethyl sulfoxide (DMSO) was then added to the wells at concentrations of 0, 10, 20, 40, 60, 80, and 100 μM, followed by 24-hour incubation. Cell viability was assessed using the Cell Counting Kit-8 (CCK-8) assay according to the manufacturer’s protocol. Absorbance was measured at 450 nm using a microplate reader, with cell viability calculated as: cell viability %=[(ODm-ODb)/(ODc-ODb)]*100 % (ODs: The OD value of measure group； ODc: The OD value of control group; ODb: The OD value of blank group).

Silymarin was used as a positive control. The supernatant of HepG2 cells was collected from different experimental groups and separated by centrifugation (4 °C, 1000 rpm, 15 minutes). The levels of ALT and AST in the supernatant were determined following the manufacturer's instructions provided with the commercial assay kits. The optical density (OD) values of each well were measured at a wavelength of 510 nm using a microplate reader. These values are expressed in units per liter (U/L) or millimoles per liter (mmol/L).

The cell culture supernatant was collected and separated by centrifugation at 4°C and 1000 rpm for 15 minutes. Levels of IL-4, IL-10, and TNF-α in the supernatant were determined using enzyme-linked immunosorbent assay (ELISA), strictly following the manufacturer’s protocols provided with the corresponding commercial ELISA kits. These values were quantified in picograms per milliliter (pg/mL) based on the standard curves generated.

Test compounds were prepared via 3-fold serial dilution (10 concentrations, 10 μM starting) with *R*(−)-deprenyl as positive control, with technical replicates. Compounds (100×final concentration) in 384-well source plates were transferred as 200 nL aliquots to assay plates. Min/Max controls received 200 nL 100% DMSO. All wells except Min received 10 μL 10 nM MAO-B (Min: buffer), centrifuged (1000 × g, 1 min), and incubated at 25°C for 15 min. After adding 10 μL 0.5 μM substrate, plates were centrifuged, incubated protected from light (60 min), then stopped with 20 μL stop solution, centrifuged, and equilibrated (20 min). Fluorescence was measured (microplate reader) and quantified (pg/mL) against a standard curve.

**Statistical analysis***.* Data are presented as mean ± standard deviation (S.D.). Student’s t-test was used for comparisons between two groups, while one-way analysis of variance (ANOVA) was performed for multiple group comparisons. Statistical significance was defined as P < 0.05.

# Experimental details, ^1^H, ^13^C NMR, HRMS and HPLC spectra


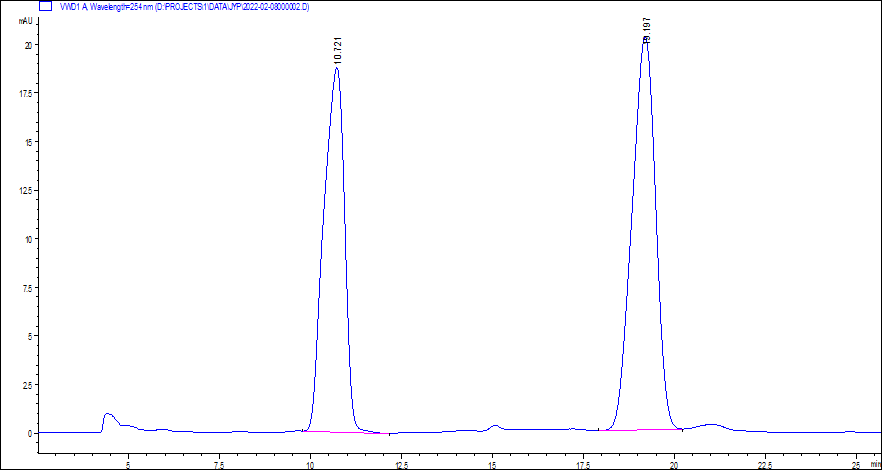
**Fig. S1.** The HPLC separation chromatogram of **1** on a chiral column.


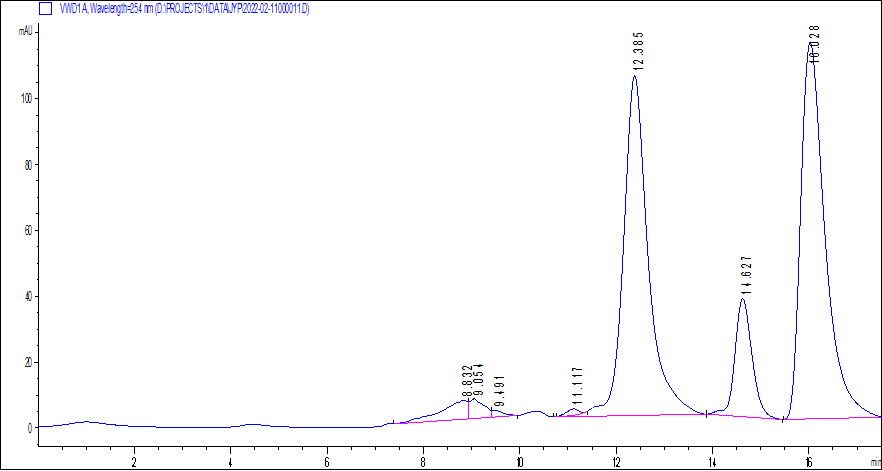


**Fig. S2.** The HPLC separation chromatogram of **2** on a chiral column.


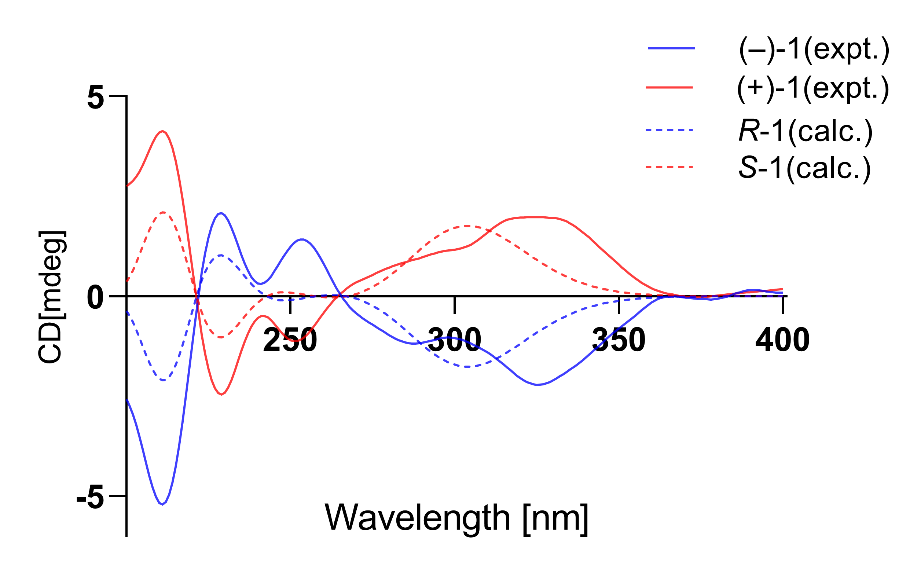


**Fig. S3.** The experimental ECD spectra of compounds (–)-**1** and (+)-**1** in MeOH and the calculated ECD Spectra of 1ʹ*S*-**1** and 1ʹ*R*-**1**.


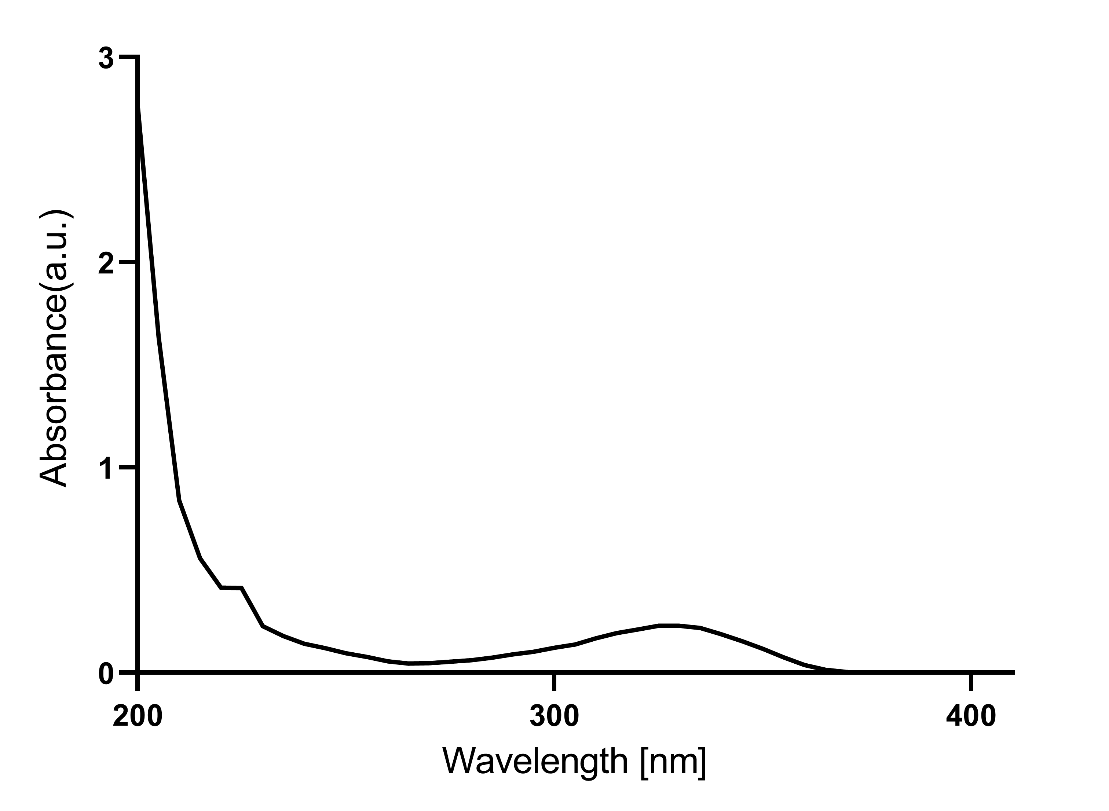


**Fig. S4.** The UV spectrum of compound **1**.

**
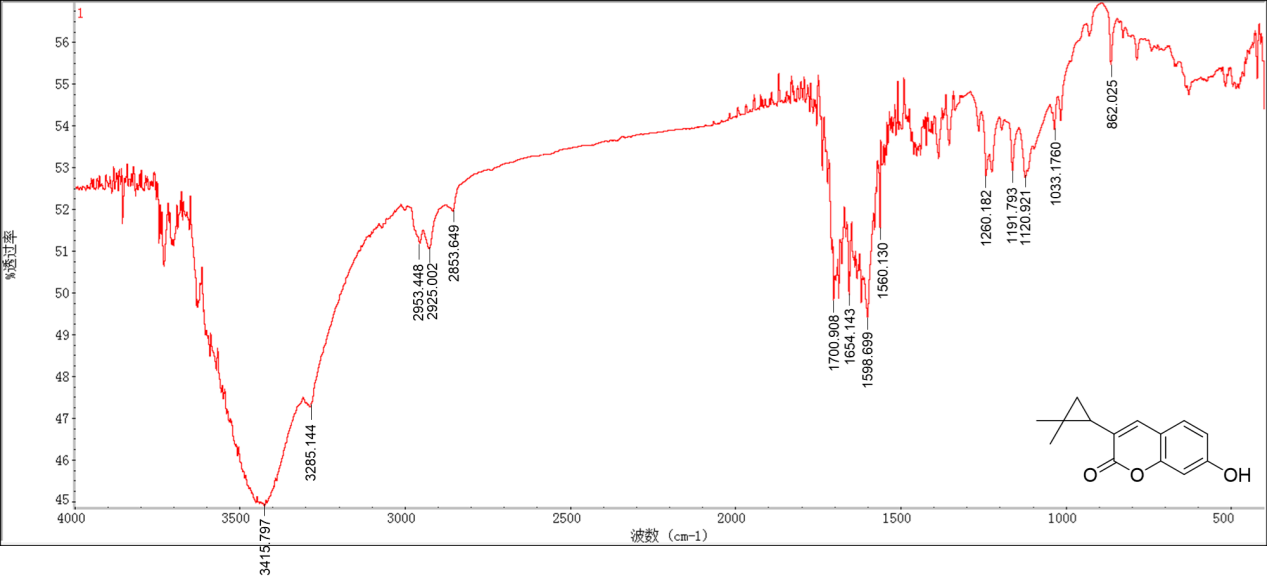
**

**Fig. S5.** The IR spectrum of compound **1.**


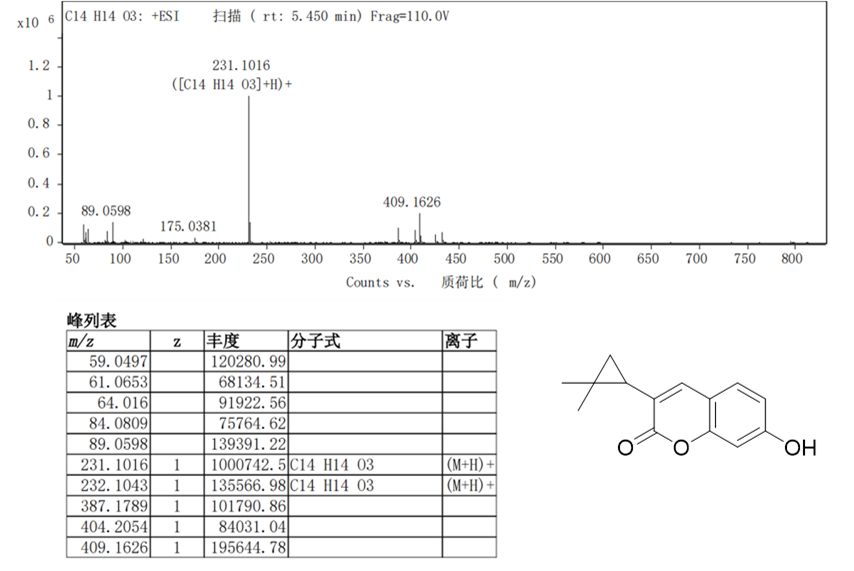


**Fig. S6.** The (+)-HR-ESI-MS of compound **1**.


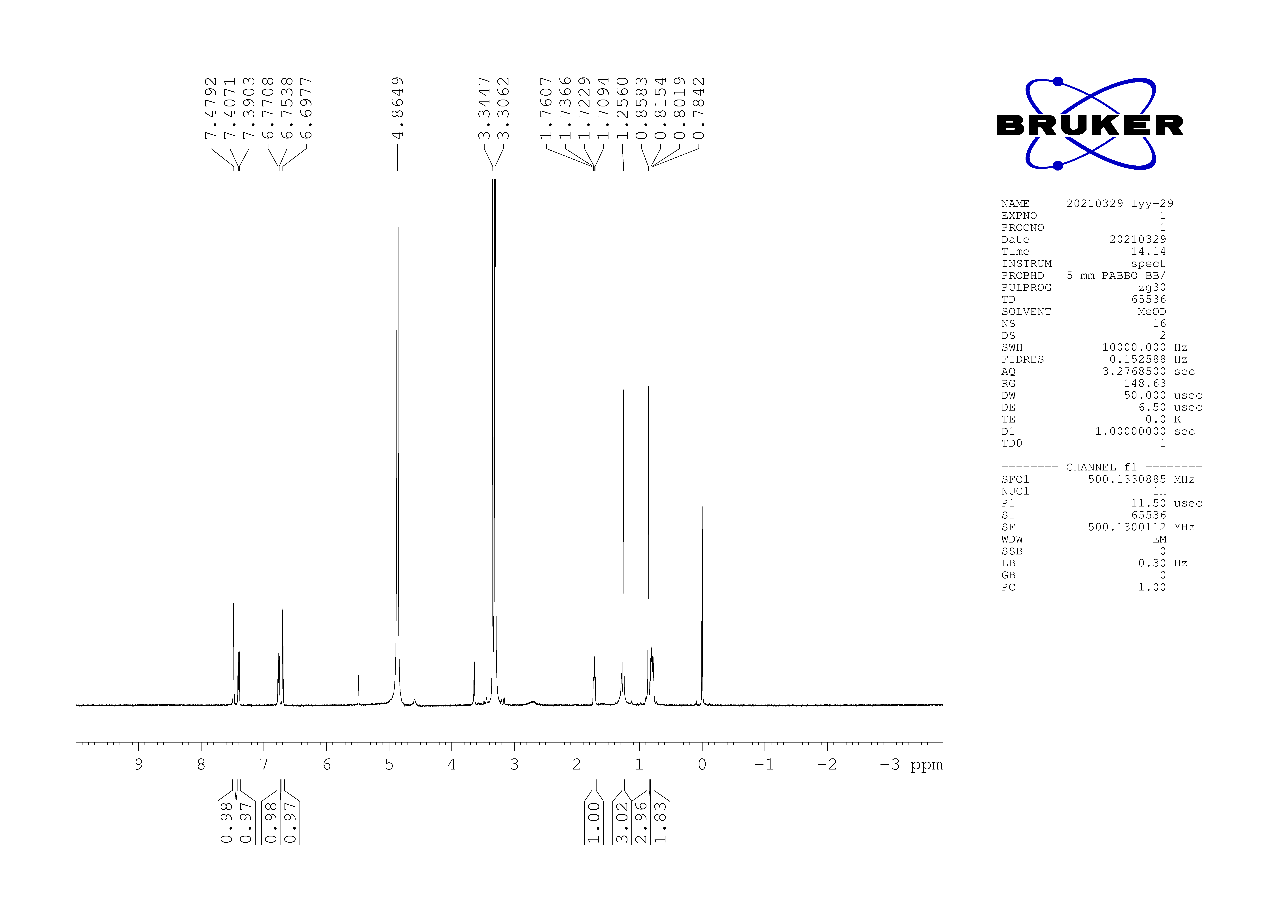
**Fig. S7** The ^1^H NMR spectrum of compound **1** in CD_3_OD at 500 M.


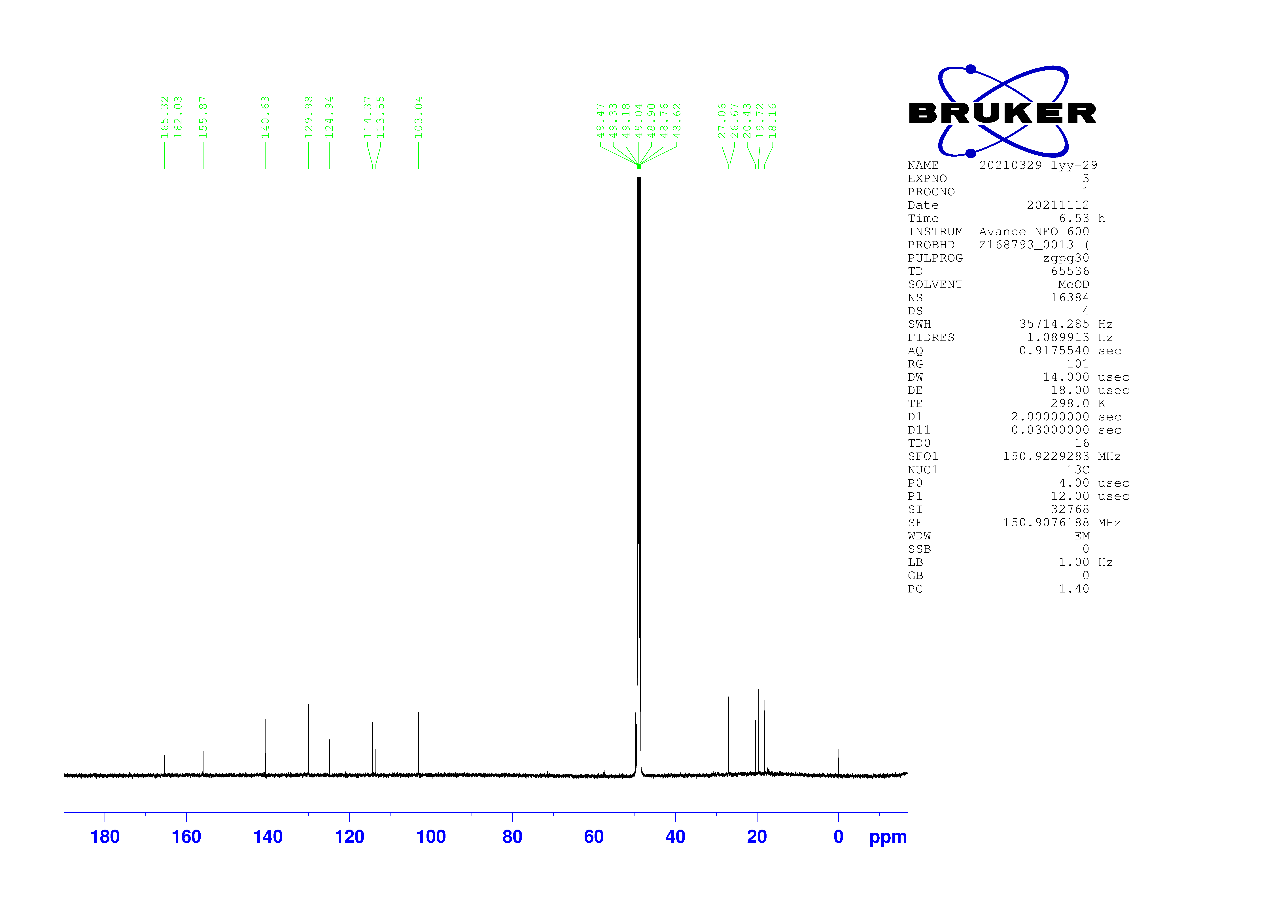
**Fig. S8.** The ^13^C NMR spectrum of compound **1** in CD_3_OD at 125 M.


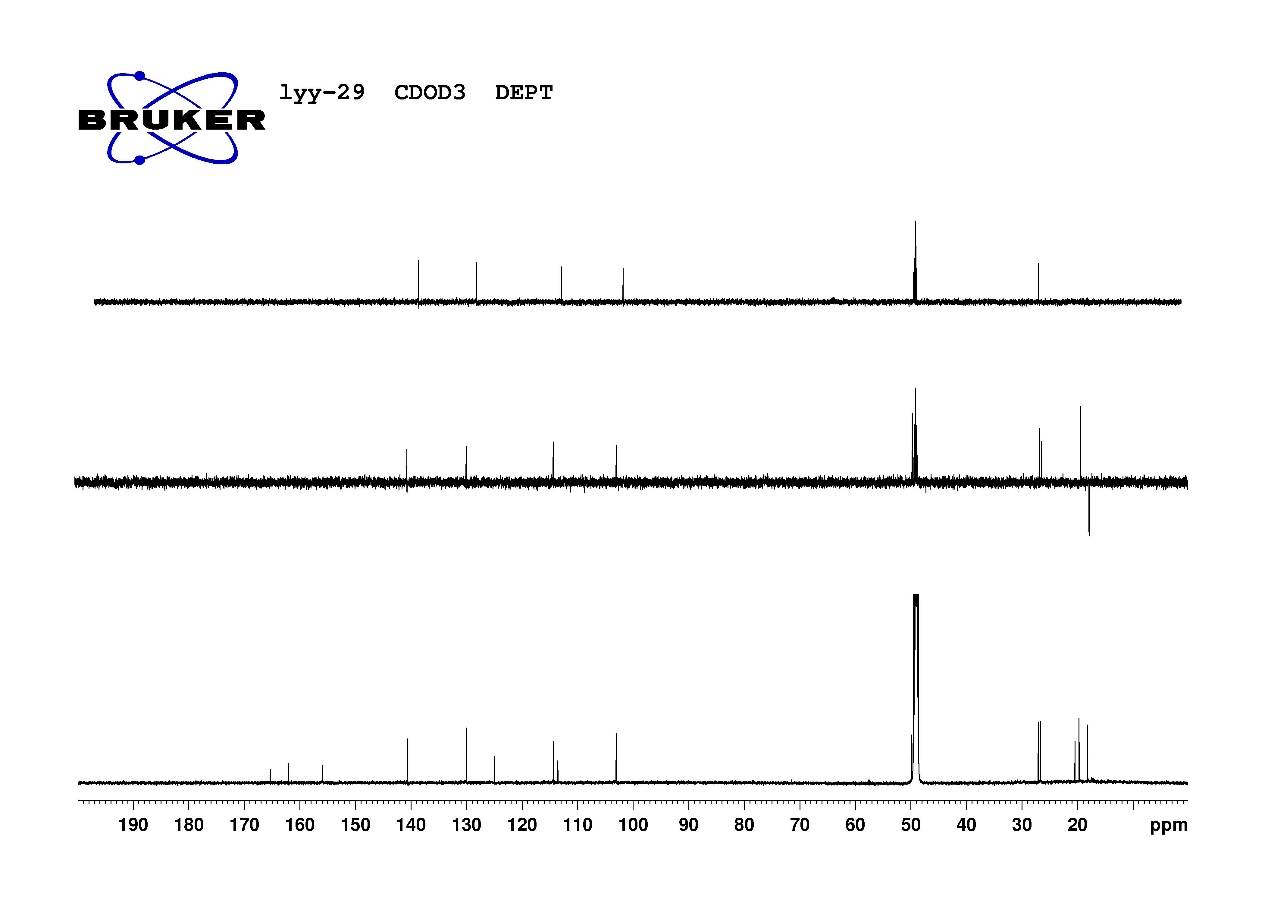


**Fig. S9.** The DEPT spectrum of compound **1** in CD_3_OD at 150 M.


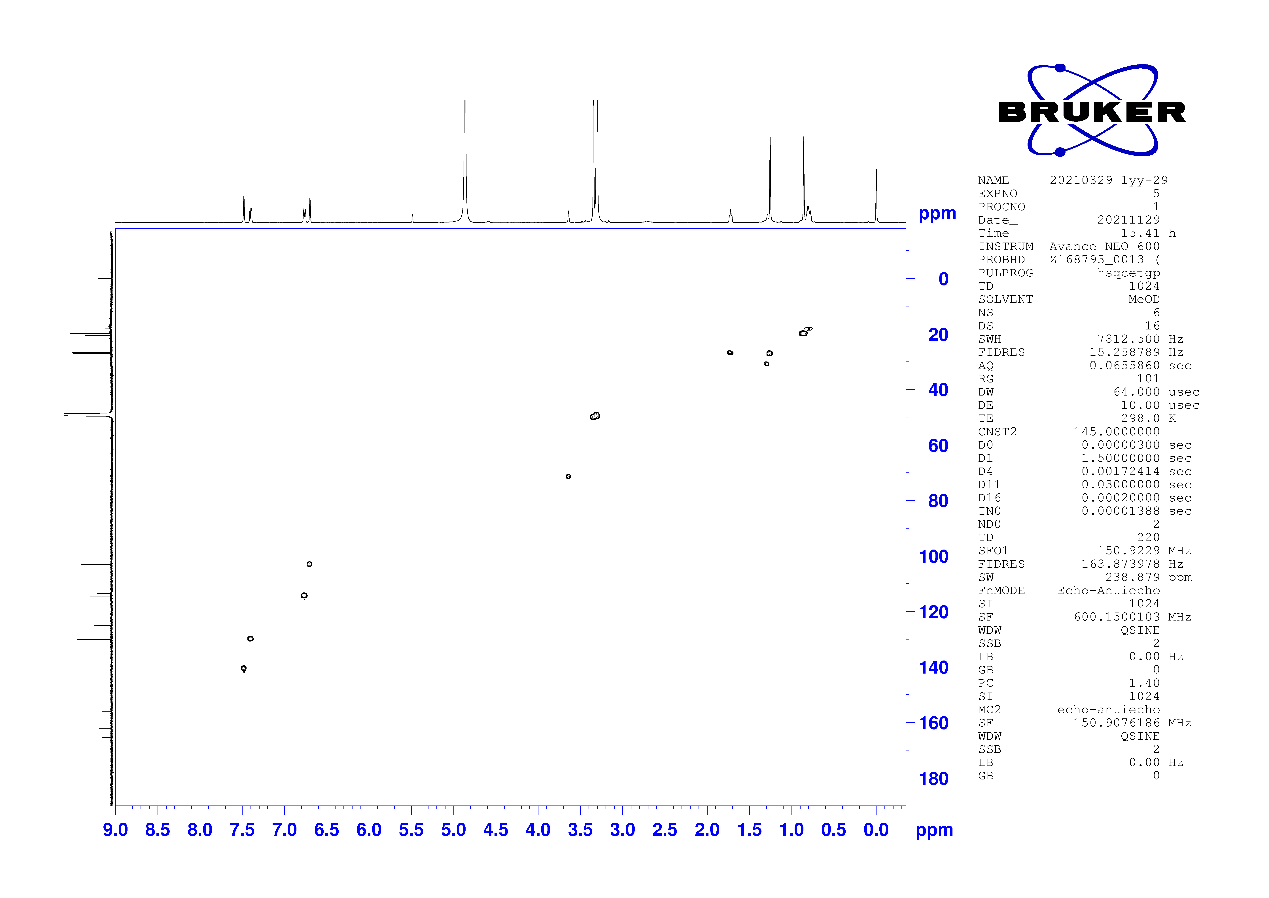


**Fig. S10.** The HSQC spectrum of compound **1** in CD_3_OD at 600 M.


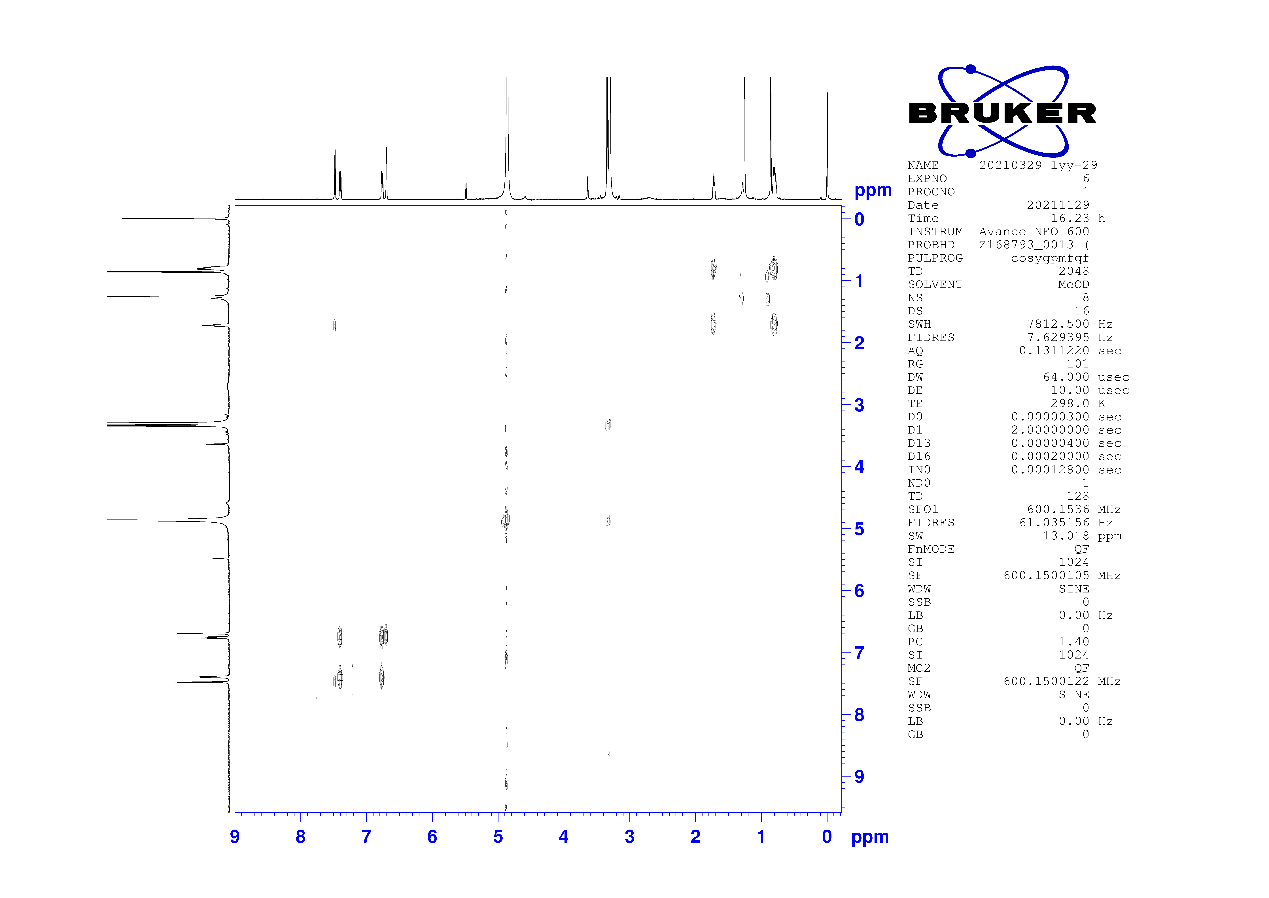


**Fig. S11.** The ^1^H-^1^H COSY spectrum of compound **1** in CD_3_OD at 600 M.


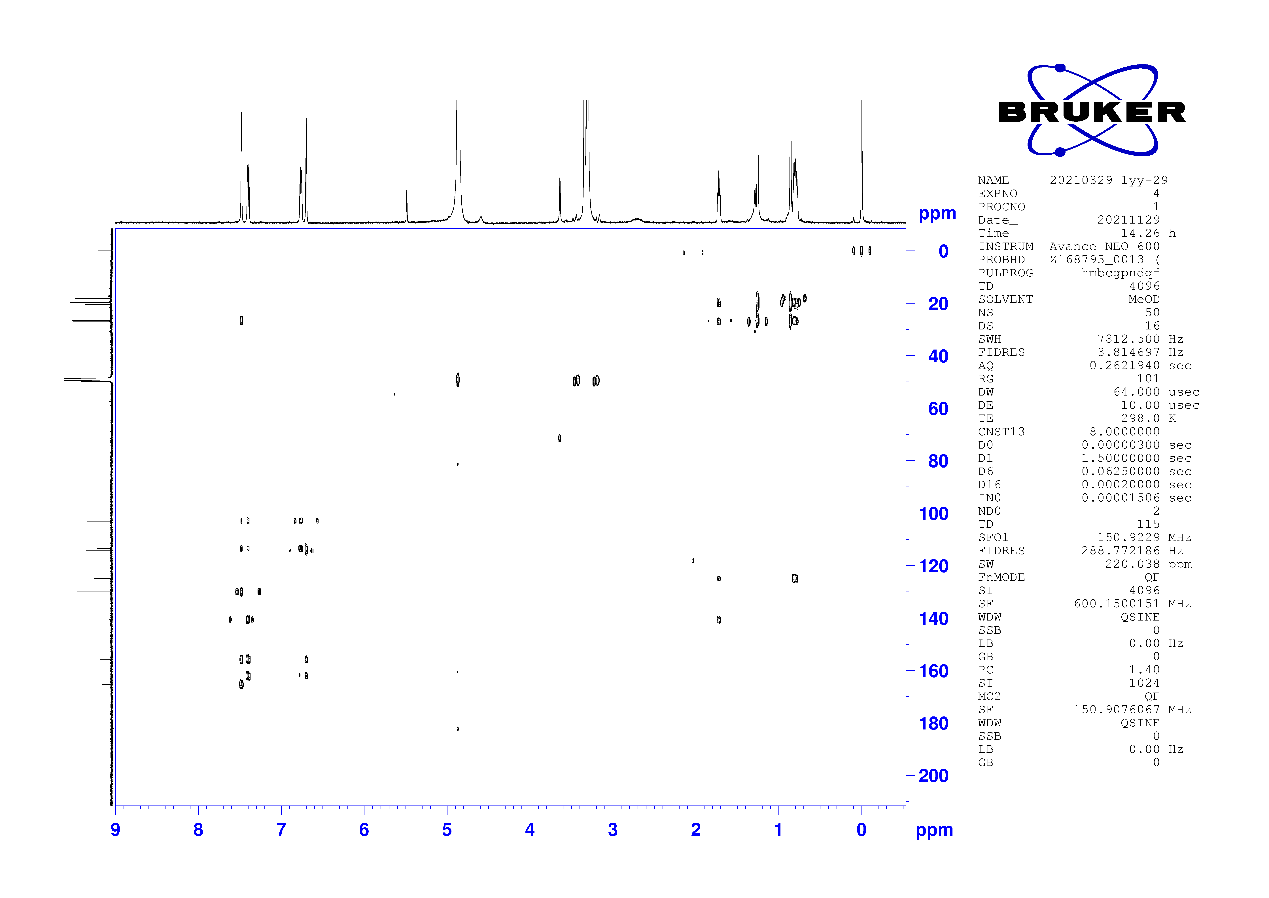


**Fig. S12.** The HMBC spectrum of compound **1** in CD_3_OD at 600 M.


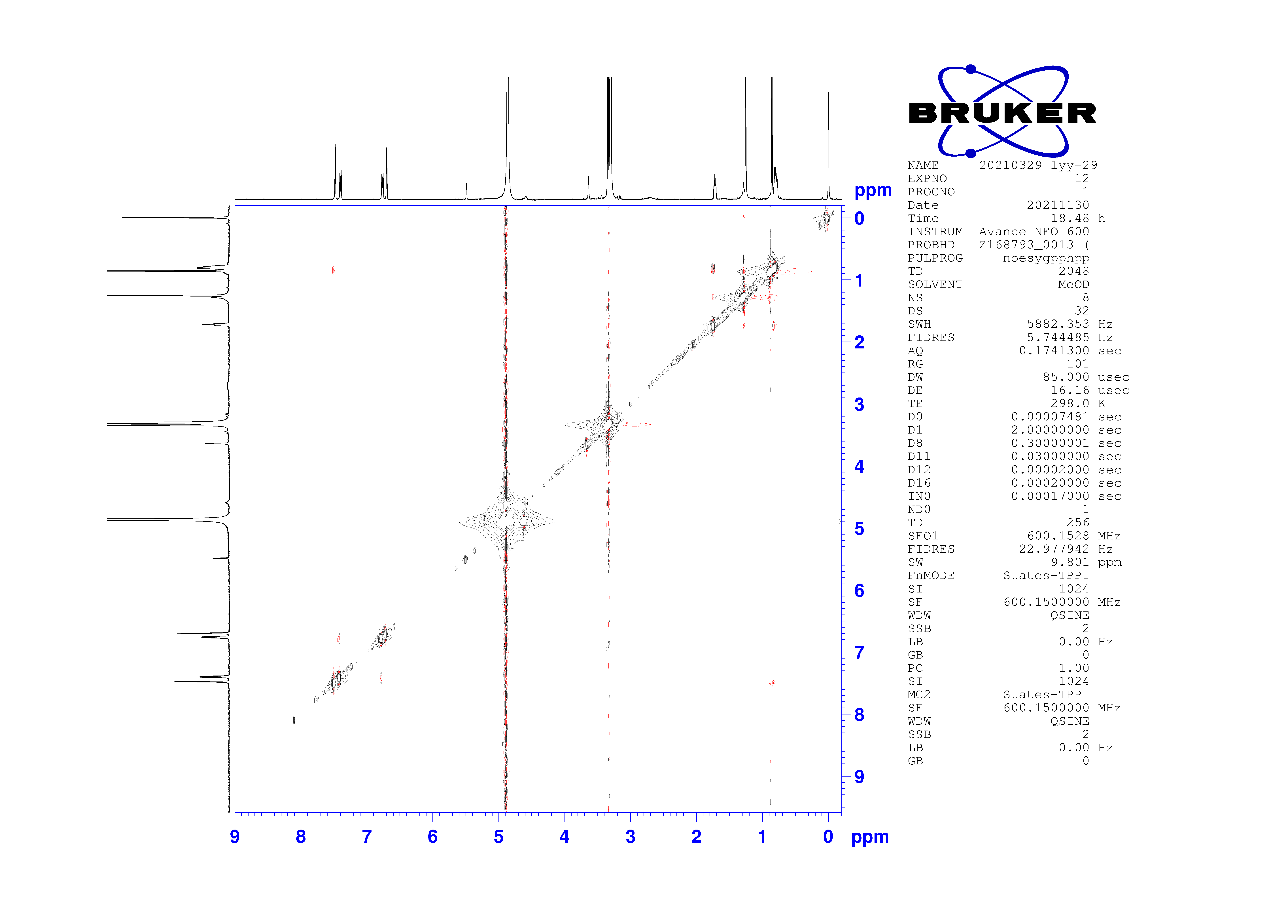


**Fig. S13.** The NOESY spectrum of compound **1** in CD_3_OD at 600 M.


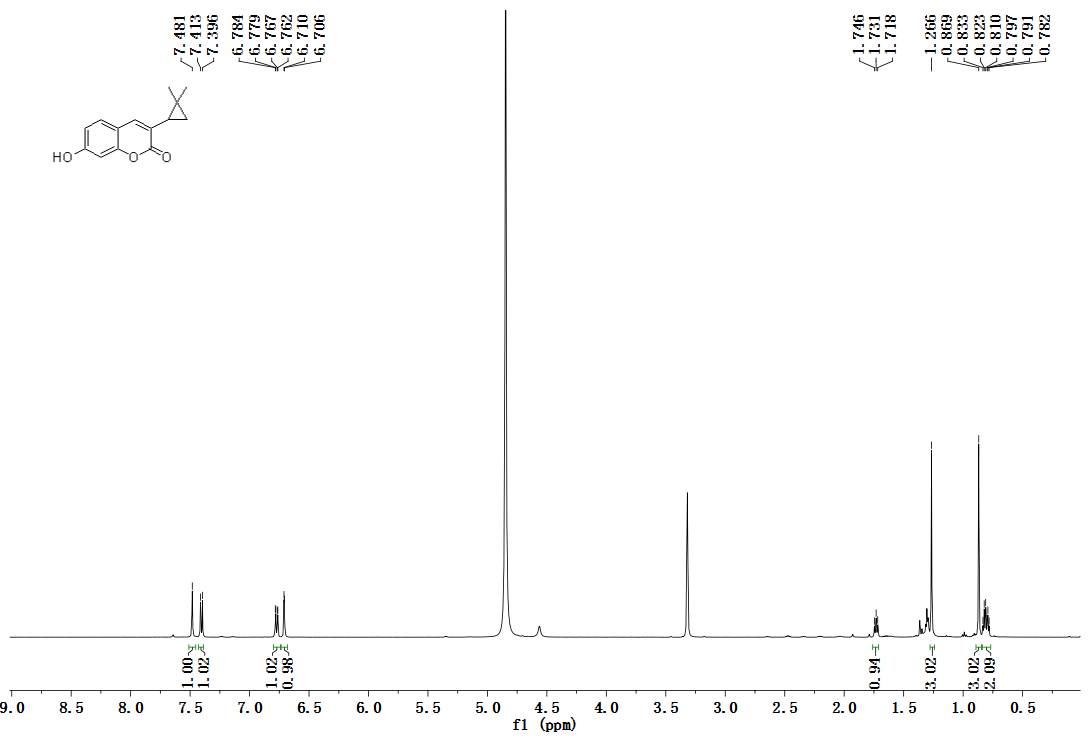


**Fig. S14.** The ^1^H NMR spectrum of synthetic compound **1** in CD_3_OD.


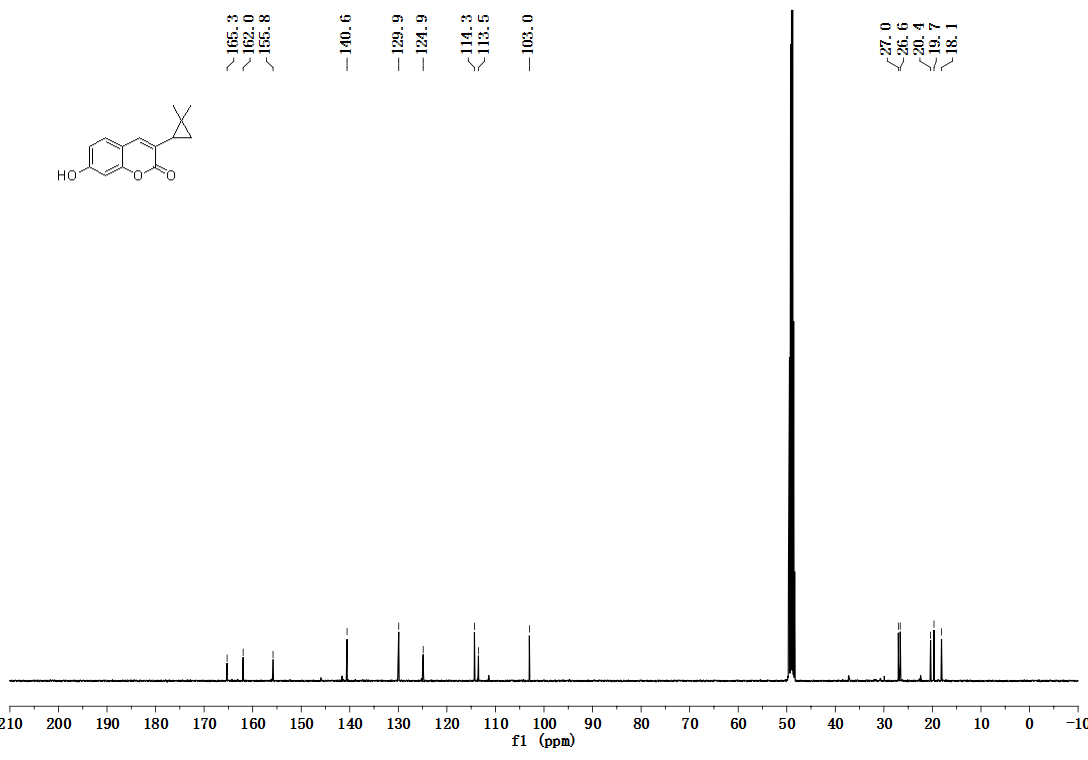


**Fig. S15.** The ^13^C NMR spectrum of synthetic compound **1** in CD_3_OD.


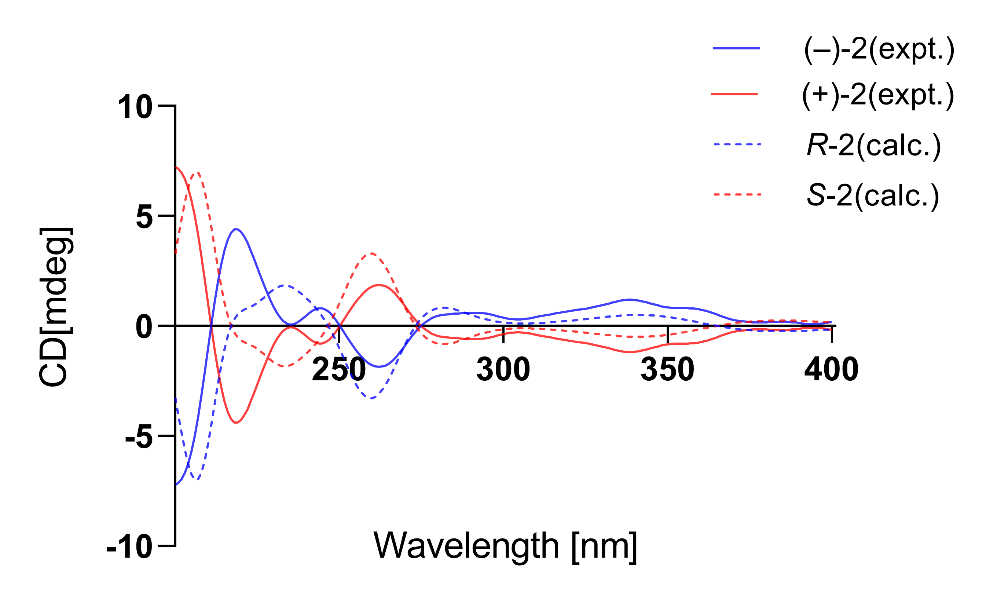


**Fig. S16.** The experimental ECD spectra of compounds (+)-**2** and (–)-**2** in MeOH.


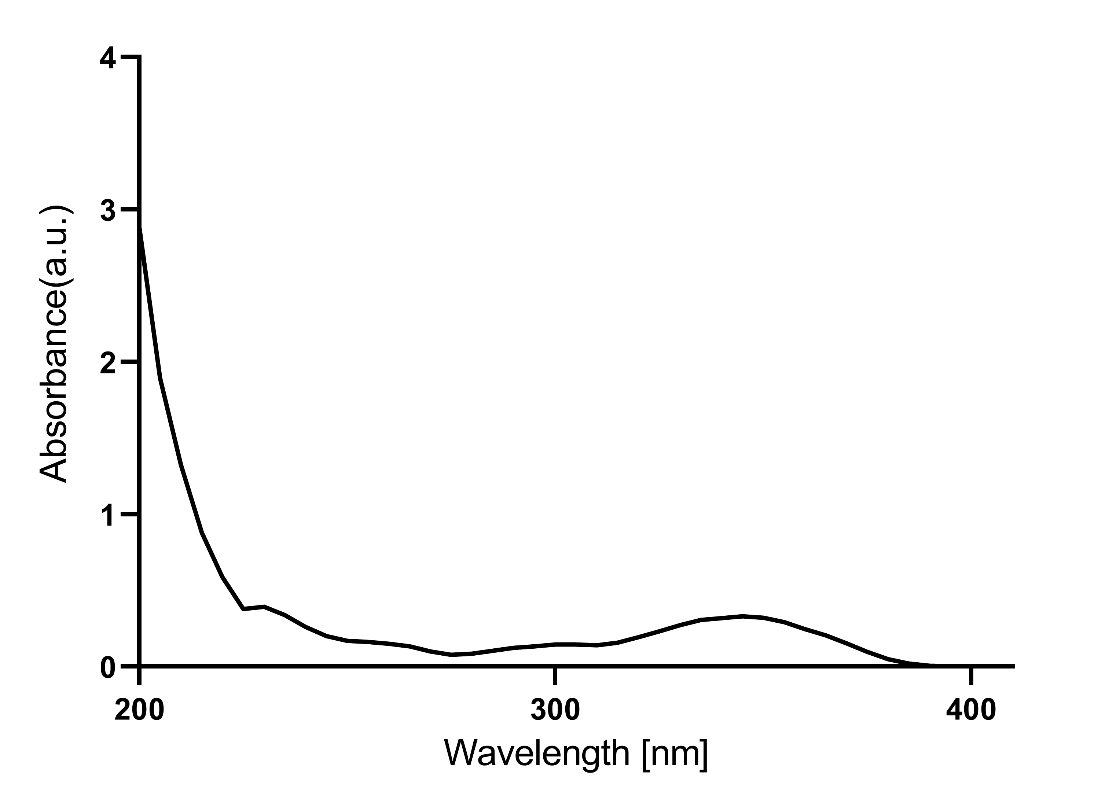


**Fig. S17.** The UV spectrum of compound **2**.


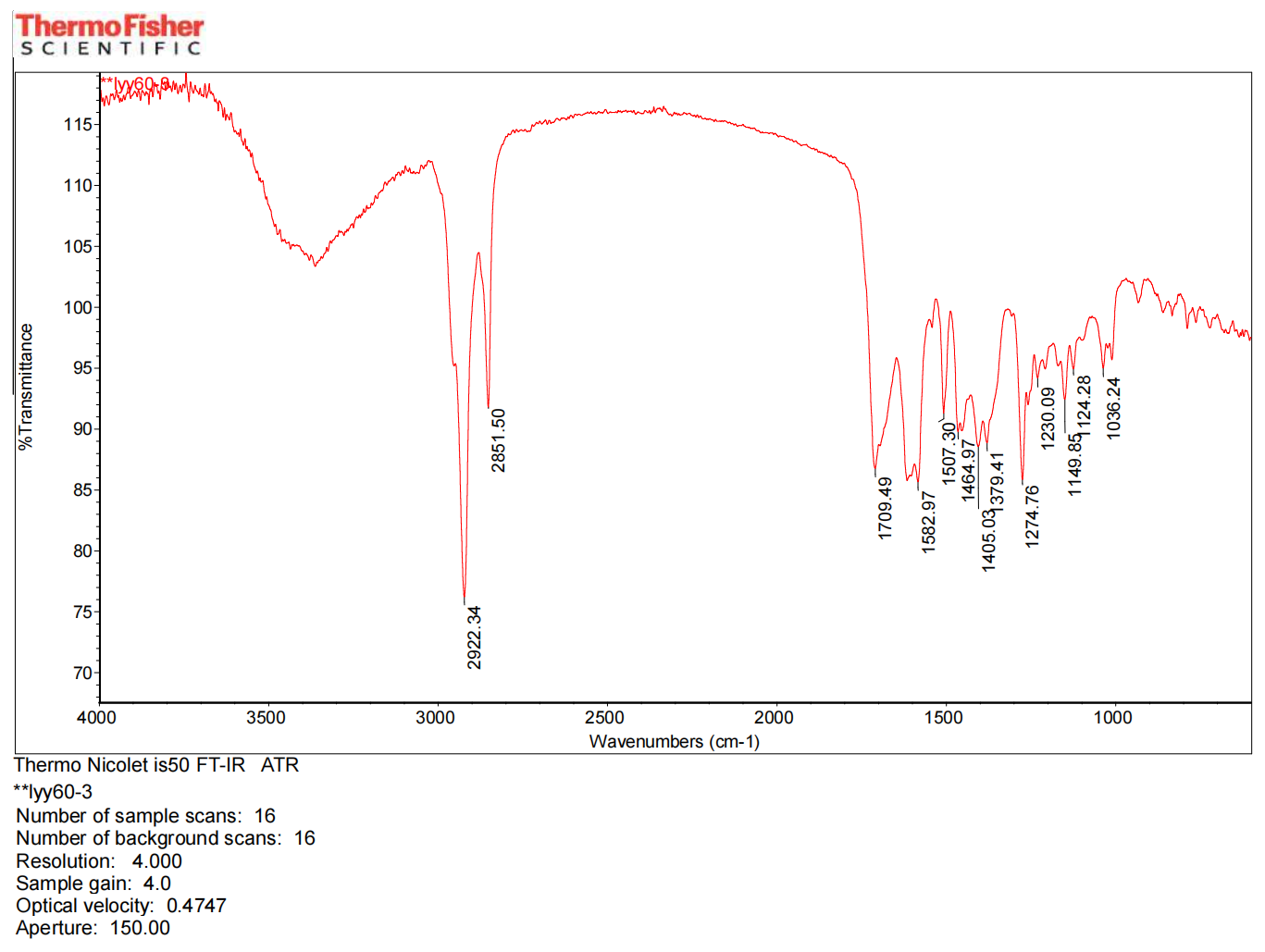


**Fig. S18.** The IR spectrum of compound **2.**


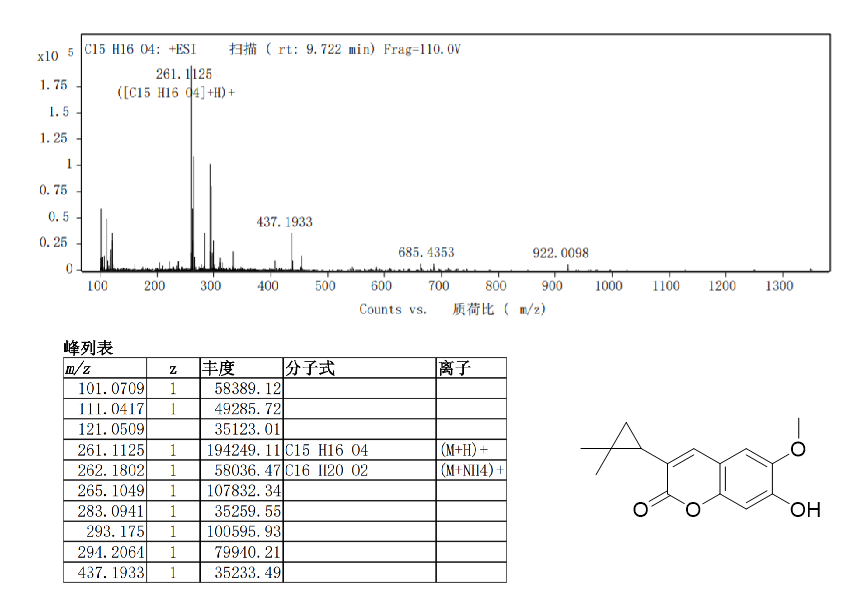


**Fig. S19.** The (+)-HR-ESI-MS of compound **2**.


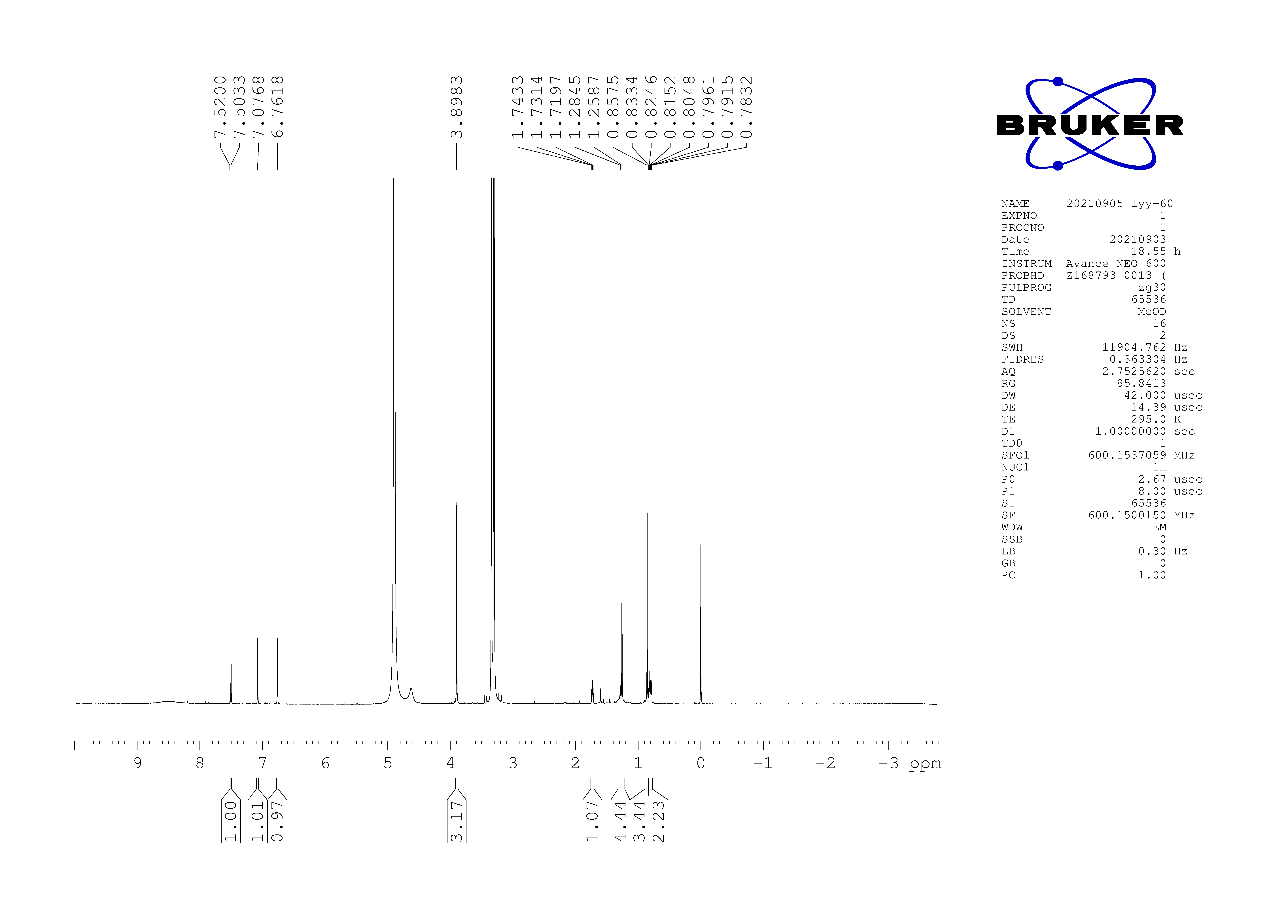


**Fig. S20.** The ^1^H NMR spectrum of compound **2** in CD_3_OD at 600 M.





**Fig. S21.** The ^13^C NMR spectrum of compound **2** in CD_3_OD at 150 M.


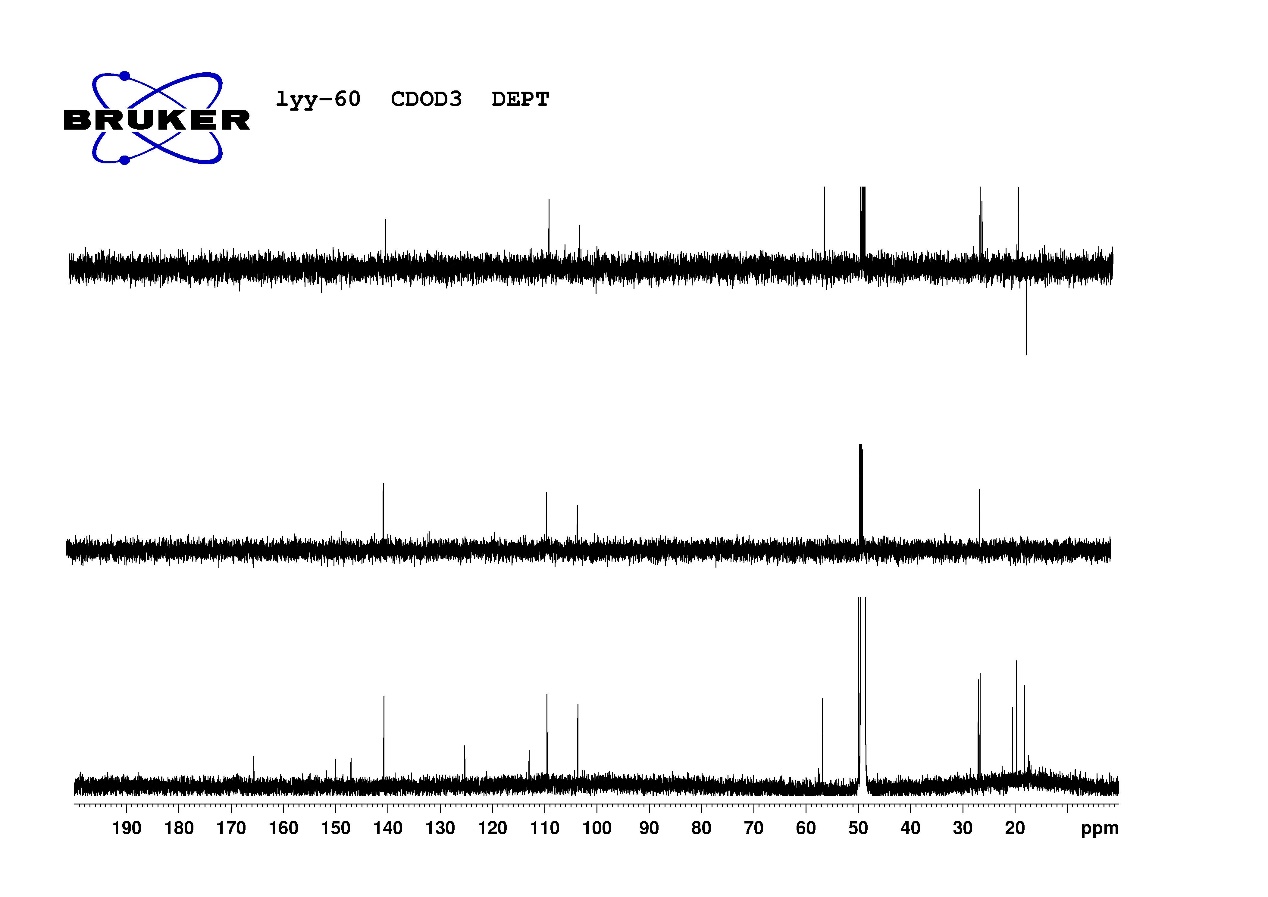


**Fig. S22.** The DEPT spectrum of compound **2** in CD_3_OD at 150 M.


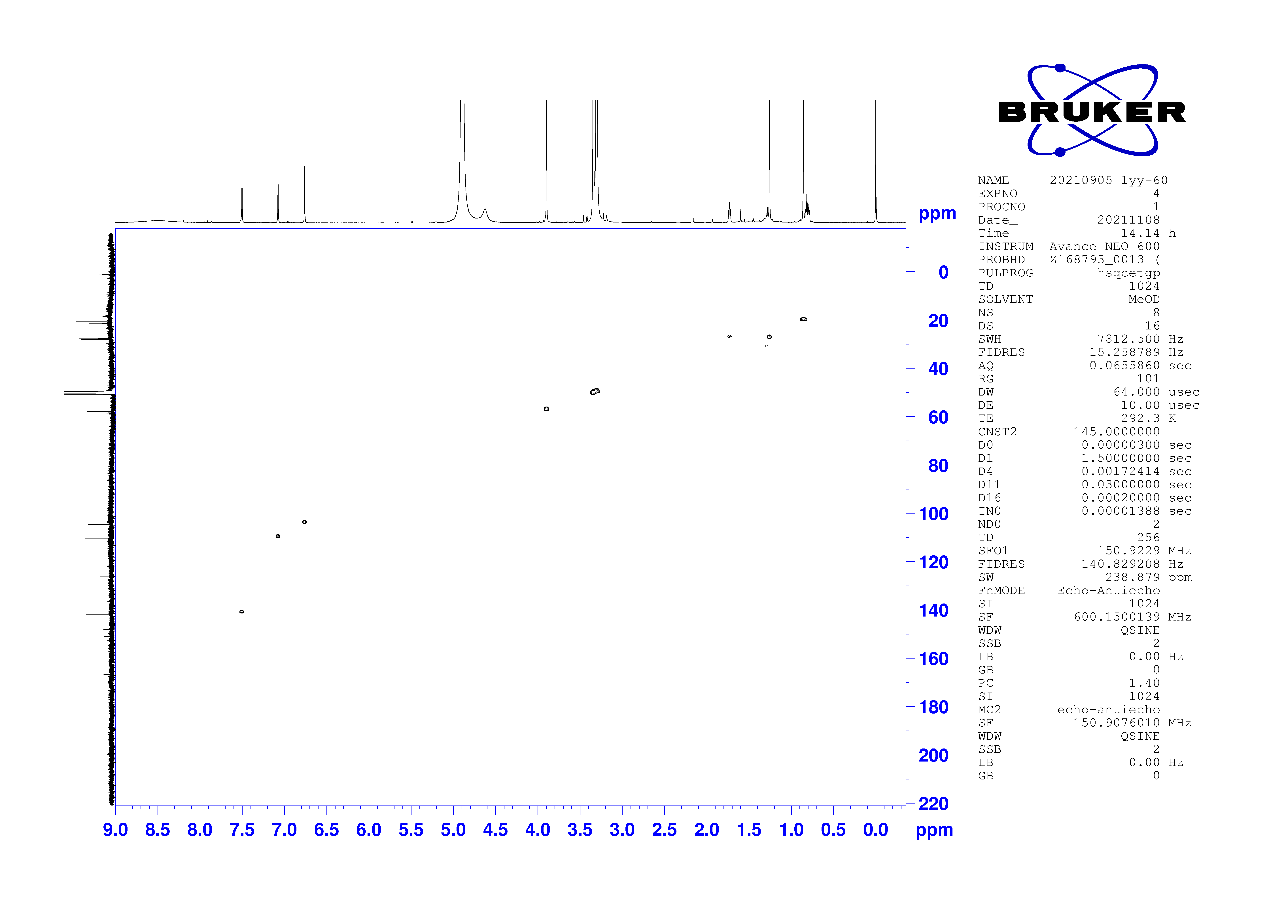


**Fig. S23.** The HSQC spectrum of compound **2** in CD_3_OD at 600 M.


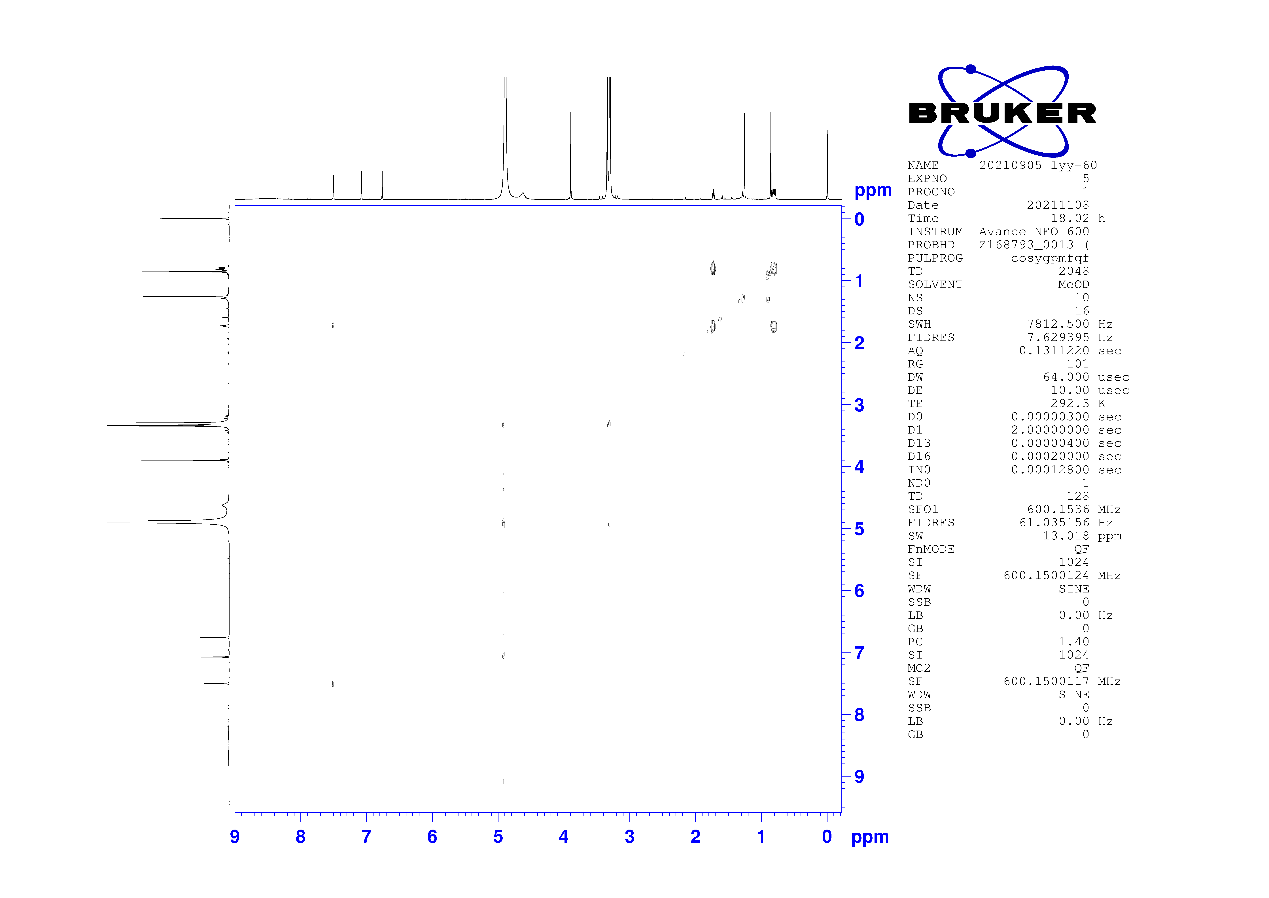


**Fig. S24.** The ^1^H-^1^H COSY spectrum of compound **2** in CD_3_OD at 600 M.


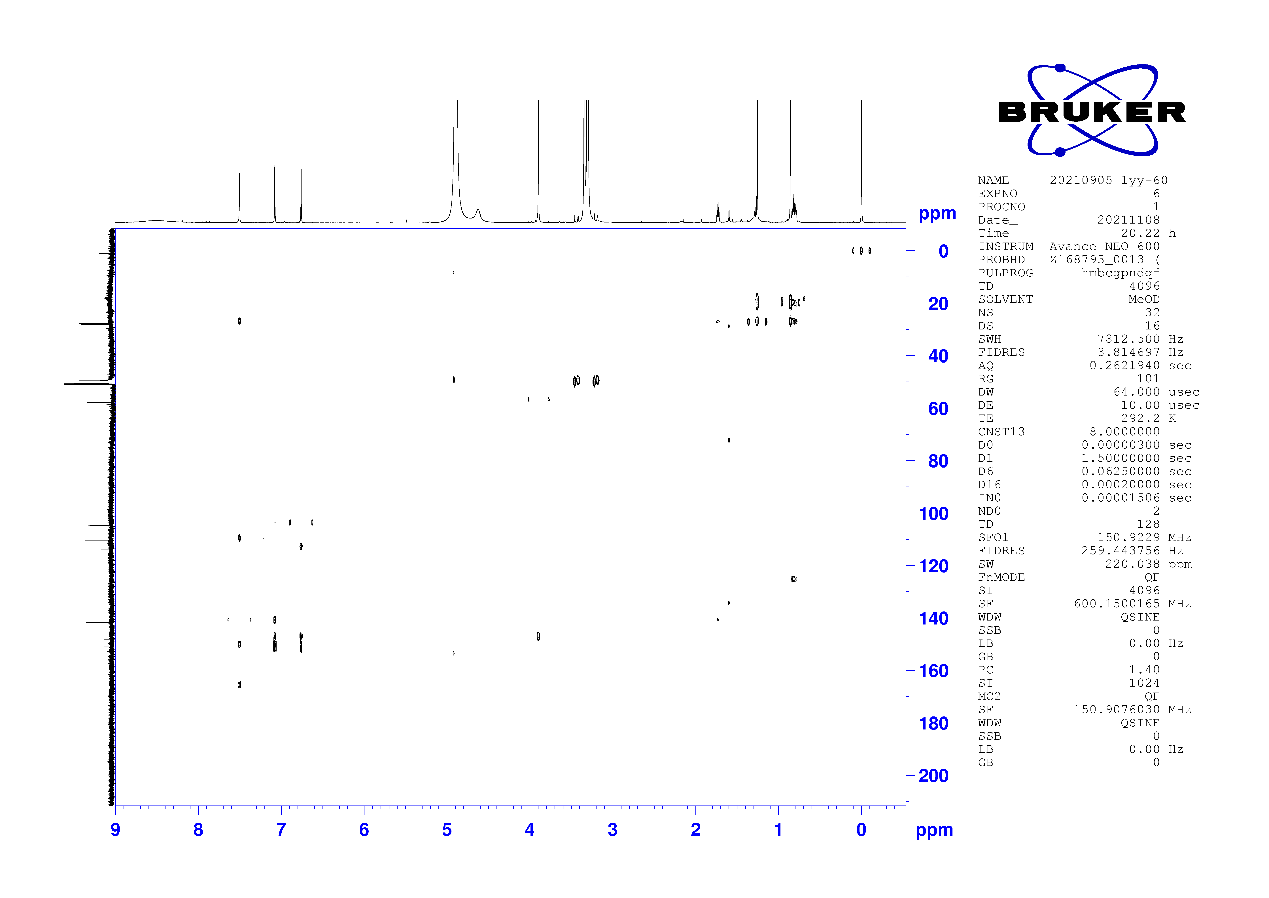


**Fig. S25.** The HMBC spectrum of compound **2** in CD_3_OD at 600 M.


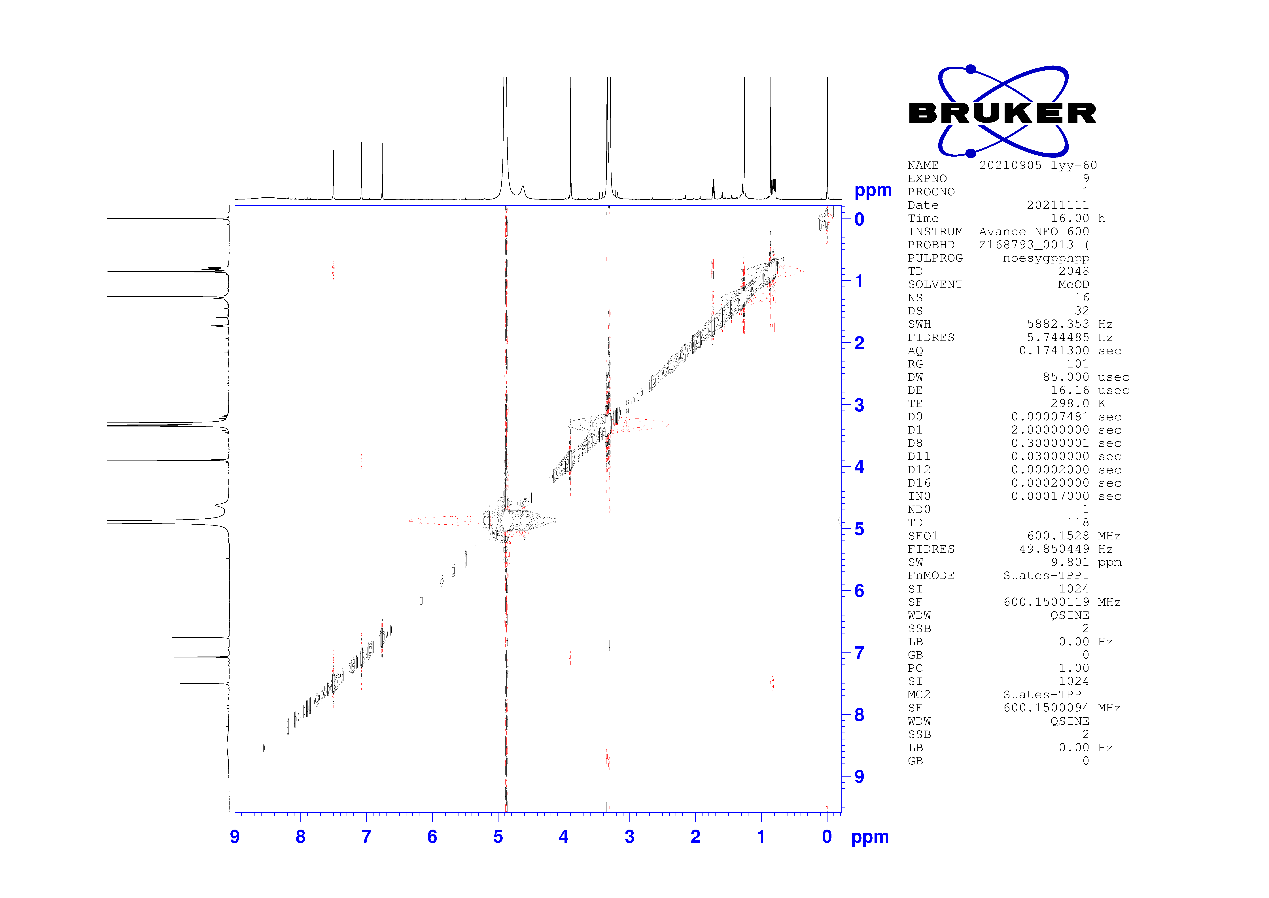


**Fig. 26.** The NOESY spectrum of compound **2** in CD_3_OD at 600 M.


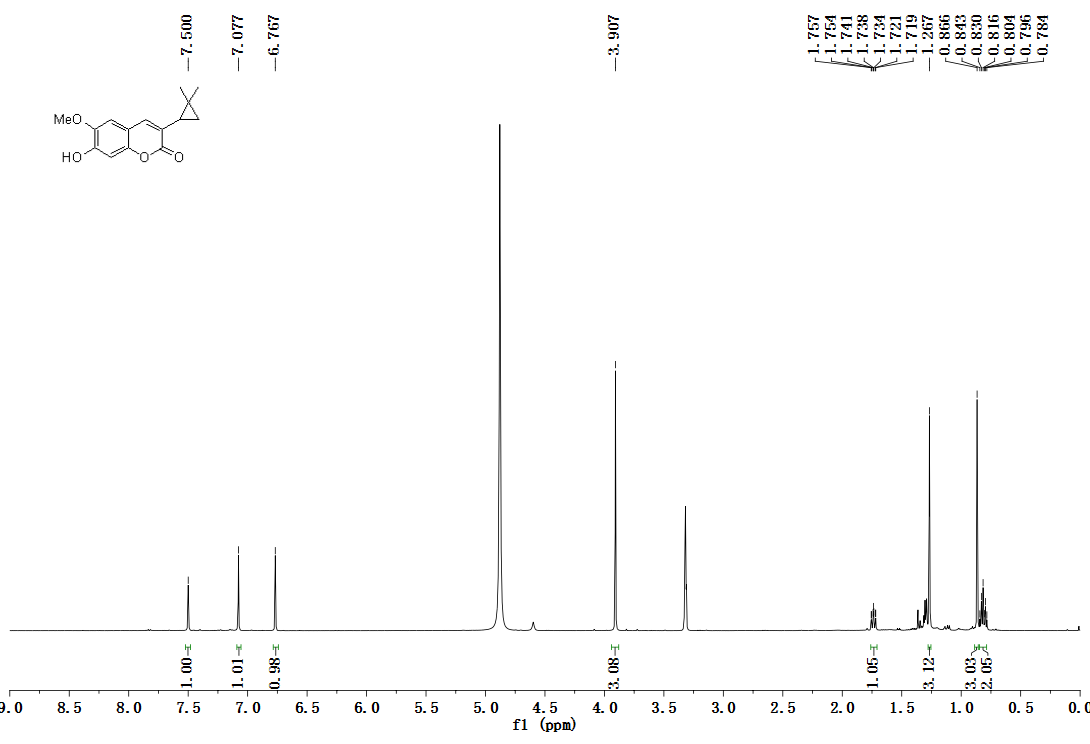


**Fig. S27.** The ^1^H NMR spectrum of synthetic compound **2** in CD_3_OD.


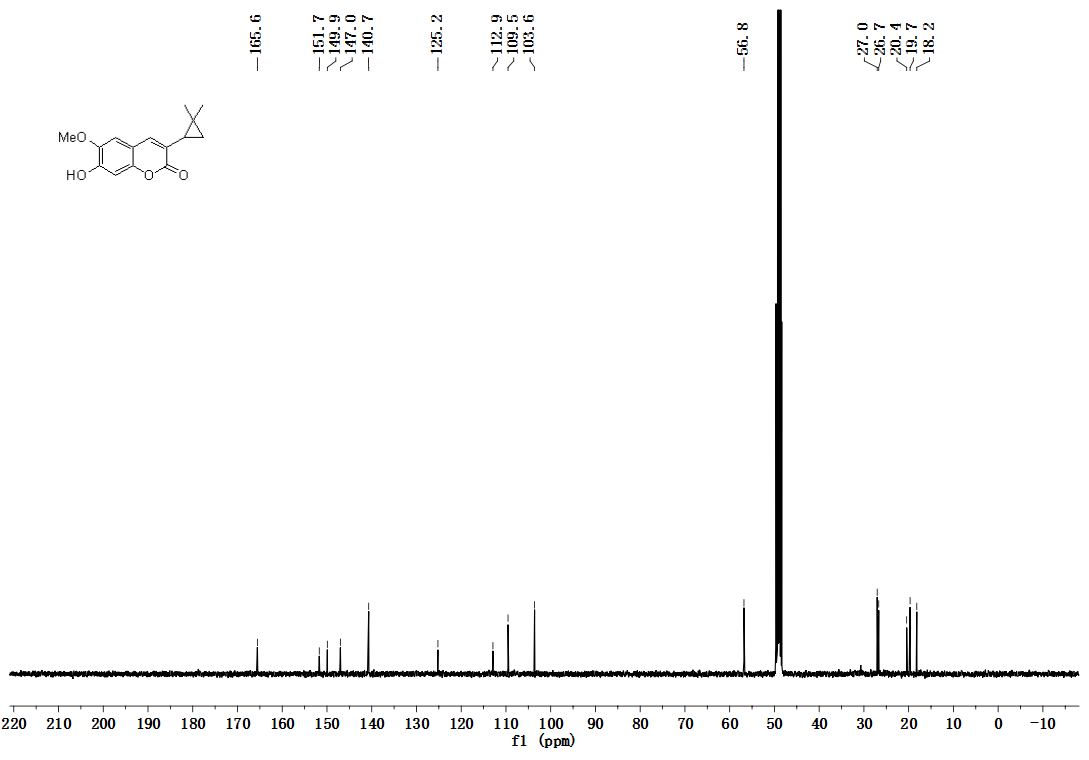


**Fig. S28.** The ^13^C NMR spectrum of synthetic compound **2** in CD_3_OD.

1. [↑](#footnote-ref-1)
2. [↑](#footnote-ref-2)
